# Malmquist productivity index for two-stage network systems under data uncertainty: A real-world case study

**DOI:** 10.1371/journal.pone.0307277

**Published:** 2024-07-18

**Authors:** Seyed Ehsan Shojaie, Seyed Jafar Sadjadi, Reza Tavakkoli-Moghaddam

**Affiliations:** 1 Department of Industrial Engineering, Science and Research Branch, Islamic Azad University, Tehran, Iran; 2 School of Industrial Engineering, Iran University of Science and Technology, Tehran, Iran; 3 School of Industrial Engineering, College of Engineering, University of Tehran, Tehran, Iran; National Kaohsiung University of Science and Technology, TAIWAN

## Abstract

The measurement of productivity change in decision-making units (DMUs) is crucial for assessing their performance and supporting efficient decision-making processes. In this paper, we propose a new approach for measuring productivity change using the Malmquist productivity index (MPI) within the context of two-stage network data envelopment analysis (TSNDEA) under data uncertainty. The two-stage network structure represents a realistic model for DMUs in various fields, such as insurance companies, bank branches, and mutual funds. However, traditional DEA models do not adequately address the issue of data uncertainty, which can significantly impact the accuracy of productivity measurements. To address this limitation, we integrate the MPI methodology with an uncertain programming framework to tackle data uncertainty in the productivity change measurement process. Our proposed approach enables the evaluation of productivity change by capturing both technical efficiency and technological progress over time. By incorporating fuzzy mathematical programming into the DEA framework, we model the inherent uncertainty in input and output data more effectively, enhancing the robustness and reliability of productivity measurements. The utilization of the proposed approach provides decision-makers with a comprehensive analysis of productivity change in DMUs, allowing for better identification of efficiency improvements or potential areas for enhancement. The findings from our study can enhance the decision-making process and facilitate more informed resource allocation strategies in real-world applications.

## 1. Introduction

Efficiency and productivity assessments are crucial for organizations seeking continuous improvement and growth in today’s highly competitive and dynamic business environment [1–3]. Various methodologies and techniques have been developed to evaluate the performance of decision-making units (DMUs) in different areas and applications such as agriculture, banking, communication, education, energy, finance, fishery, forestry, healthcare, insurance, manufacturing, power, supply chain, transportation, and tourism [4–6]. One widely used approach is the data envelopment analysis (DEA), which measures the relative efficiency of DMUs based on their input-output characteristics [7–9]. However, traditional and conventional DEA models may not be suitable for evaluating the performance of complex systems characterized by two-stage networks and data uncertainty [10–14].

In recent years, researchers have recognized the importance of extending DEA models to analyze the productivity change in two-stage network systems [15–17]. Two-stage networks refer to interconnected systems with intermediate processes or stages between the input and output stages. These systems are prevalent in real-world case studies and applications such as airlines, bank branches, electricity distribution companies, food supply chain, healthcare supply chain, insurance companies, manufacturing companies, mutual funds, research and development projects, and universities, where multiple stages are involved in the production or service delivery process [18–20]. The presence of intermediate stages in these networks allows for more efficient resource allocation, coordination of activities, and optimization of overall system performance. By understanding and analyzing the dynamics of two-stage networks, researchers can identify bottlenecks, improve process efficiency, and enhance overall system productivity.

The Malmquist productivity index (MPI) is a well-known DEA-based tool used to measure productivity change over time [21–23]. It calculates the changes in efficiency and technical progress for individual DMUs and aggregates them at the industry or country level. However, the traditional MPI does not account for data uncertainty, which is inherent in real-world situations and problems [24–26]. Data uncertainty and ambiguity arise due to various sources, including measurement errors, missing data, and imprecise estimates. Ignoring data uncertainty can lead to biased and unreliable productivity assessments. Hence, there is a need to develop effective productivity measurement techniques that consider data uncertainty in the context of two-stage network systems.

To address this research gap, this paper proposes the application of credibility-based fuzzy network data envelopment analysis (CFNDEA) approach integrated with Malmquist productivity index to evaluate the productivity change of two-stage network systems under data uncertainty. Fuzzy network DEA models extend the traditional DEA framework by incorporating fuzzy set theory to handle imprecision and vagueness in input-output data [27–29]. Credibility theory, on the other hand, deals with uncertainty by assigning credibility weights to observations based on their accuracy and reliability [30–32]. The objectives of this research paper are threefold: I) to develop a fuzzy network DEA approach for evaluating the efficiency and productivity change of two-stage network DMUs; II) to integrate credibility theory into the fuzzy network DEA model to account for data uncertainty; and III) to apply the proposed methodology to a real case study involving Iranian mutual funds.

Mutual funds are investment vehicles that pool money from multiple investors to invest in a diversified portfolio of securities such as stocks, bonds, or a combination of both [33–35]. They are managed by professional fund managers who make investment decisions on behalf of the investors. Mutual funds offer individual investors access to a wide range of investment opportunities with the potential for diversification and professional management. Investors can buy shares or units of mutual funds, which represent their ownership in the fund’s assets. The mutual fund’s performance is typically measured by its net asset value (NAV), which is the value of the fund’s assets minus its liabilities, divided by the number of outstanding shares or units. Investors can buy and sell mutual fund shares at the fund’s NAV, which is calculated at the end of each trading day.

Performance evaluation of mutual funds is important as it helps investors assess the fund’s historical returns, risk levels, and consistency. It aids in comparing different funds and making informed investment decisions. Evaluating performance also helps investors track fund managers’ skills and determine if the fund aligns with their financial goals and risk tolerance [36–38]. The proposed fuzzy network DEA model, integrated with credibility theory, will enable us to evaluate the productivity change of the mutual funds network under data uncertainty. By considering imprecise and unreliable data, the model will provide more robust and accurate performance assessments. It should be explained that the main advantages of the current research can be summarized as follows:

I) Proposing a new Malmquist productivity index that is capable of being used for network systems in the presence of uncertain data. II) Measuring efficiency score, efficiency change, technological change, and productivity change of two-stage DMUs under fuzzy panel data. III) The linearity of the proposed credibility-based fuzzy network DEA approach. IV) The unique efficiency decomposition of two-stage DMUs under data ambiguity. V) The discriminatory power of the credibility-based fuzzy network model exceeds that of a classical network DEA model. VI) The proposed uncertain network Malmquist productivity index is applied for the dynamic performance evaluation of mutual funds. VII) Identifying the state of productivity of mutual funds both in general and in terms of sub-units during two consecutive time periods. VIII) The sensitivity analysis of results from the proposed CFNDEA approach and uncertain network MPI is examined under different confidence levels.

The remainder of this research paper is organized as follows: Section 2 provides a two-stage network DEA approach based on additive efficiency decomposition. Section 3 delineates the classification of the literature review and underscores significant gaps in the existing literature. Section 4 presents the proposed methodology, including the formulation of the fuzzy network DEA model and the integration of credibility theory. Section 5 proposes a new Malmquist productivity index for two-stage network systems under data uncertainty. Section 6 outlines the case study design and data collection process as well as the results and discussion. Finally, Section 7 introduces the conclusion and future research directions.

## 2. Literature review

In this section, an extensive review of the literature is conducted from two distinct perspectives: theoretical (focusing on network DEA and fuzzy chance-constrained programming) and practical (applying network DEA in the context of mutual funds). Furthermore, the study outlines the identified gaps in the existing literature that serve as the focal point of this research. Table 1 summarizes the main characteristics of the fuzzy chance-constrained network DEA researches considering different aspects such as uncertain measure types, including possibility (POS), necessity (NEC), credibility (CR), and general fuzzy (GF). Moreover, a nuanced categorization of mutual fund performance assessment through network DEA approach is depicted comprehensively in Table 2.

**Table 1:** Network data envelopment analysis and fuzzy chance-constrained programming: A literature review.

Year	Research	Network DEA Modeling Approach	Uncertain Measure				Data Structure		Dynamic Performance Assessment Approach	Application
			POS	NEC	CR	GF	Static	Dynamic		
2013	Bai-Qing et al. [39]	Relational Efficiency Decomposition	✓				**✓**		**-**	Commercial Banks
2017	Yousefi et al. [40]	Multi-Objective Programming	**✓**				**✓**		**-**	Sustainable Supply Chains
2017	Zhou et al. [41]	Additive Efficiency Decomposition	**✓**				**✓**		**-**	Commercial Banks
2018	Peykani & Mohammadi [42]	Production Possibility Set	**✓**				**✓**		**-**	Investment Firms
2018	Zhou et al. [43]	Slacks-Based Measure			**✓**		**✓**		**-**	Industrial Production and Environmental Management Systems
2019	Nasseri & Khatir [44]	Multiplicative Efficiency Decomposition	**✓**				**✓**		**-**	Bank Branches
2019	Nosrat et al. [45]	Additive Efficiency Decomposition			**✓**		**✓**		**-**	Non-Life Insurance Companies
2020	Roghaee et al. [46]	Additive Efficiency Decomposition		**✓**			**✓**		**-**	Electricity Companies
2021	Chen & Xu [47]	Multi-Objective Programming	**✓**				**✓**		**-**	Green Supply Chains
2021	Peykani et al. [48]	Additive Efficiency Decomposition				**✓**	**✓**		**-**	Investment Firms
2023	Pourbabagol et al. [49]	Slacks-Based Measure	**✓**	**✓**			**✓**		**-**	Agile Supply Chains
The Current Research		Additive Efficiency Decomposition			**✓**			**✓**	Malmquist Productivity Index	Mutual Funds

**Table 2:** Network data envelopment analysis and mutual funds: A literature review.

Year	Research	Network DEA Modeling Approach	Data Type		Uncertain Programming Approach	Data Structure		Dynamic Performance Assessment Approach
			Crisp	Uncertain		Static	Dynamic	
2012	Premachandra et al. [50]	Additive Efficiency Decomposition	**✓**		**-**	**✓**		**-**
2016	Galagedera et al. [51]	Additive Efficiency Decomposition	**✓**		**-**	**✓**		**-**
2016	Premachandra et al. [52]	Additive Efficiency Decomposition	**✓**		**-**	**✓**		**-**
2017	Sánchez-González et al. [53]	Slacks-Based Measure	**✓**		**-**	**✓**		**-**
2018	Galagedera et al. [54]	Additive Efficiency Decomposition	**✓**		**-**	**✓**		**-**
2019	Galagedera [55]	Additive Efficiency Decomposition	**✓**		**-**	**✓**		**-**
2020	Hsieh et al. [56]	Slacks-Based Measure	**✓**		**-**	**✓**		**-**
2020	Galagedera et al. [57]	Production Possibility Set	**✓**		**-**	**✓**		**-**
2020	Tsolas [58]	Independent	**✓**		**-**	**✓**		**-**
2021	Fukuyama & Galagedera [59]	Production Possibility Set	**✓**		**-**	**✓**		**-**
2022	Peykani et al. [60]	Non-Cooperative Game		**✓**	Robust Optimization	**✓**		**-**
The Current Research		Additive Efficiency Decomposition		**✓**	Fuzzy Chance-Constrained Programming		**✓**	Malmquist Productivity Index

As evident in Tables 1 and 2, the goal of this research is to fill the current research gap in the literature by introducing a novel and efficient Malmquist productivity index for mutual funds operating within two-stage network systems under data uncertainty. To achieve this goal, the research utilizes the concepts of the Malmquist productivity index, network data envelopment analysis approach, additive efficiency decomposition technique, credibility theory, and fuzzy chance-constrained programming. Finally, the applicability and efficacy of the proposed uncertain network Malmquist productivity index are demonstrated through a real case study involving Iranian mutual funds.

## 3. The network DEA approach: Additive efficiency decomposition

**Fig 1:**
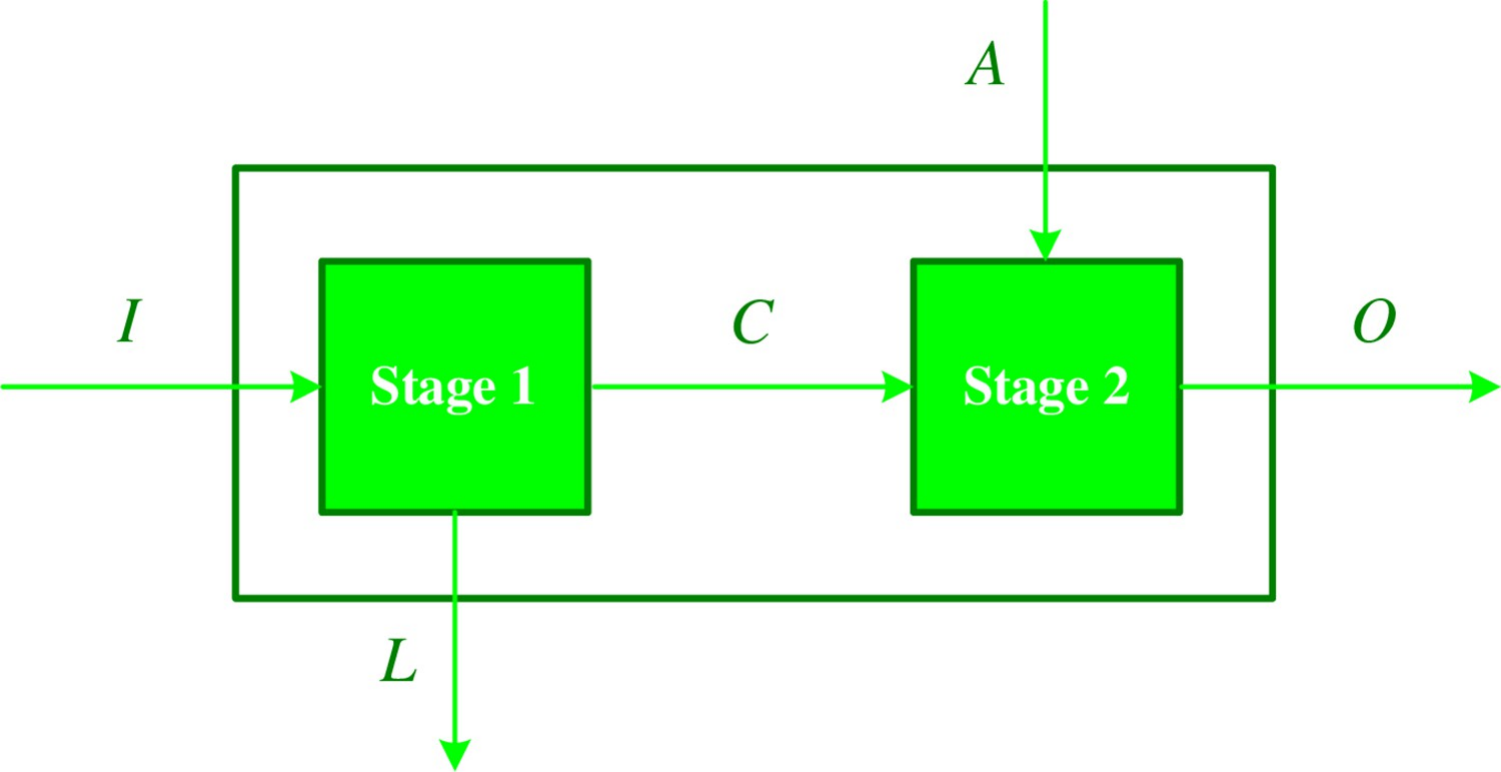
The graphical presentation of general two-stage structure.

Fig 1 visually depicts a generic two-stage framework, comprising a collection of *S* homogeneous decision-making units DMU_*s*_ (*s* = 1,…,*S*). In the initial stage, each DMU possesses *P* inputs *I*_*ps*_ (*p* = 1,…,*P*), while *Y* outputs *L*_*ys*_ (*y* = 1,…,*Y*) (referred to as leakage variables) exit the system. Additionally, *G* intermediate variables *C*_*gs*_ (*g* = 1,…*G*) establish a connection between the first and second stages. Moving on to the second stage, there are *X* additional inputs *A*_*xs*_(*x* = 1,…,*X*) and ultimately *Q* outputs *Q*_*qs*_ (*q* = 1,…,*Q*). It should be explained that the non-negative weights *α*_*p*_ (*p* = 1,…,*P*), *λ*_*y*_ (*y* = 1,…,*Y*), *ω*_*g*_ (*g* = 1,…,*G*), *μ*_*x*_ (*x* = 1,…,*X*), and *β*_*q*_ (*q* = 1,…,*Q*) are assigned to the *I*_*ps*_ (*p* = 1,…,*P*), *L*_*ys*_ (y = 1,…,*Y*), *C*_*gs*_ (*g* = 1,…,*G*), *A*_*xs*_ (*x* = 1,…,*X*), and *Q*_*qs*_ (*q* = 1,…,*Q*), respectively.

The present study utilizes the additive efficiency decomposition approach as a fundamental nonparametric network DEA method for evaluating the performance of both the overall system and its sub-components (stages 1 and 2). This approach proves to be suitable for analyzing a two-stage structure with a leakage variable in the first stage and additional inputs in the second stage [61]. It is worth mentioning that the additive efficiency decomposition approach holds a prominent position among the various methods employed in the field of network data envelopment analysis [62]. The subsequent section provides a detailed explanation of the modeling process utilizing an additive efficiency decomposition approach for a general two-stage structure. Referring to Fig 1, the efficiency score of the initial stage and subsequent stage for DMU_*k*_ (the DMU under investigation) can be computed using Models (1) and (2) correspondingly:

$$\Theta_{k}^{S1}=\mathrm{Max}\;\frac{\sum_{y=1}^{Y}L_{yk}\lambda_{y}+\sum_{g=1}^{G}C_{gk}\omega_{g}}{\sum_{p=1}^{P}I_{pk}\alpha_{p}}$$ (1)

$$S.t.\;\frac{\sum_{y=1}^{Y}L_{ys}\lambda_{y}+\sum_{g=1}^{G}C_{gs}\omega_{g}}{\sum_{p=1}^{P}I_{ps}\alpha_{p}}\leq 1,\;\forall s$$

$$\alpha_{p},\lambda_{y},\omega_{g}\geq 0,\;\forall p,y,g$$

$$\Theta_{k}^{S2}=\mathrm{Max}\;\frac{\sum_{q=1}^{Q}O_{qk}\beta_{q}}{\sum_{g=1}^{G}C_{gk}\omega_{g}+\sum_{x=1}^{X}A_{xk}\mu_{x}}$$ (2)

$$S.t.\;\frac{\sum_{q=1}^{Q}O_{qs}\beta_{q}}{\sum_{g=1}^{G}C_{gs}\omega_{g}+\sum_{x=1}^{X}A_{xs}\mu_{x}}\leq 1,\;\forall s$$

$$\omega_{g},\mu_{x},\beta_{q}\geq 0,\;\forall g,x,q$$

In line with the concept proposed by Chen et al. [61], the overall efficiency of the typical two-stage network procedure can be represented mathematically by Eq (3):

$$\Theta_{k}^{N}=\Psi_{k}^{S1}\Theta_{k}^{S1}+\Psi_{k}^{S2}\Theta_{k}^{S2}=\Psi_{k}^{S1}(\frac{\sum_{y=1}^{Y}L_{yk}\lambda_{y}+\sum_{g=1}^{G}C_{gk}\omega_{g}}{\sum_{p=1}^{P}I_{pk}\alpha_{p}})+\Psi_{k}^{S2}(\frac{\sum_{q=1}^{Q}O_{qk}\beta_{q}}{\sum_{g=1}^{G}C_{gk}\omega_{g}+\sum_{x=1}^{X}A_{xk}\mu_{x}})$$ (3)

Please be aware that, in Eq (3), $\Psi_{k}^{S1}$ and $\Psi_{k}^{S2}$ represent user-defined weights, which contribute to the magnitude of $\Psi_{k}^{S1}+\Psi_{k}^{S2}=1$. To elaborate, $\Psi_{k}^{S1}$ and $\Psi_{k}^{S2}$ exemplify the significance of first stage and second stage in relation to the system’s overall performance. Consequently, the efficiency score of DMU_*k*_ is determined by solving Model (4) in the ensuing manner:

$$\Theta_{k}^{N}=\mathrm{Max}\;\Psi_{k}^{S1}(\frac{\sum_{y=1}^{Y}L_{yk}\lambda_{y}+\sum_{g=1}^{G}C_{gk}\omega_{g}}{\sum_{p=1}^{P}I_{pk}\alpha_{p}})+\Psi_{k}^{S2}(\frac{\sum_{q=1}^{Q}O_{qk}\beta_{q}}{\sum_{g=1}^{G}C_{gk}\omega_{g}+\sum_{x=1}^{X}A_{xk}\mu_{x}})$$ (4)

$$S.t.\;\frac{\sum_{y=1}^{Y}L_{ys}\lambda_{y}+\sum_{g=1}^{G}C_{gs}\omega_{g}}{\sum_{p=1}^{P}I_{ps}\alpha_{p}}\leq 1,\;\forall s$$

$$\frac{\sum_{q=1}^{Q}O_{qs}\beta_{q}}{\sum_{g=1}^{G}C_{gs}\omega_{g}+\sum_{x=1}^{X}A_{xs}\mu_{x}}\leq 1,\;\forall s$$

$$\alpha_{p},\lambda_{y},\omega_{g},\mu_{x},\beta_{q}\geq 0,\;\forall p,y,g,x,q$$

As evident from Model (4), this particular model cannot be transformed into a linear program using the standard Charnes & Cooper [63] conversion technique. In order to resolve this challenge, a proposed solution by Chen et al. [61] involves defining $\Psi_{k}^{S1}$ and $\Psi_{k}^{S2}$ as Eqs (5) and (6) correspondingly:

$$\Psi_{k}^{S1}=\frac{\sum_{p=1}^{P}I_{pk}\alpha_{p}}{\sum_{p=1}^{P}I_{pk}\alpha_{p}+\sum_{g=1}^{G}C_{gk}\omega_{g}+\sum_{x=1}^{X}A_{xk}\mu_{x}}$$ (5)

$$\Psi_{k}^{S2}=\frac{\sum_{g=1}^{G}C_{gk}\omega_{g}+\sum_{x=1}^{X}A_{xk}\mu_{x}}{\sum_{p=1}^{P}I_{pk}\alpha_{p}+\sum_{g=1}^{G}C_{gk}\omega_{g}+\sum_{x=1}^{X}A_{xk}\mu_{x}}$$ (6)

Therefore, employing the aforementioned equations will lead to the transformation of Model (4) into Model (7), which can be expressed in the following manner:

$$\Theta_{k}^{N}=\mathrm{Max}\;\frac{\sum_{y=1}^{Y}L_{yk}\lambda_{y}+\sum_{g=1}^{G}C_{gk}\omega_{g}+\sum_{q=1}^{Q}O_{qk}\beta_{q}}{\sum_{p=1}^{P}I_{pk}\alpha_{p}+\sum_{g=1}^{G}C_{gk}\omega_{g}+\sum_{x=1}^{X}A_{xk}\mu_{x}}$$ (7)

$$S.t.\;\frac{\sum_{y=1}^{Y}L_{ys}\lambda_{y}+\sum_{g=1}^{G}C_{gs}\omega_{g}}{\sum_{p=1}^{P}I_{ps}\alpha_{p}}\leq 1,\;\forall s$$

$$\frac{\sum_{q=1}^{Q}O_{qs}\beta_{q}}{\sum_{g=1}^{G}C_{gs}\omega_{g}+\sum_{x=1}^{X}A_{xs}\mu_{x}}\leq 1,\;\forall s$$

$$\alpha_{p},\lambda_{y},\omega_{g},\mu_{x},\beta_{q}\geq 0,\;\forall p,y,g,x,q$$

Now, through the utilization of the Charnes & Cooper [63] conversion technique, the existing Model (7) can be transformed into a linear programming representation known as Model (8):

$$\Theta_{k}^{N}=\mathrm{Max}\;\sum_{y=1}^{Y}L_{yk}\lambda_{y}+\sum_{g=1}^{G}C_{gk}\omega_{g}+\sum_{q=1}^{Q}O_{qk}\beta_{q}$$ (8)

$$S.t.\;\sum_{p=1}^{P}I_{pk}\alpha_{p}+\sum_{g=1}^{G}C_{gk}\omega_{g}+\sum_{x=1}^{X}A_{xk}\mu_{x}=1$$

$$\sum_{y=1}^{Y}L_{ys}\lambda_{y}+\sum_{g=1}^{G}C_{gs}\omega_{g}-\sum_{p=1}^{P}I_{ps}\alpha_{p}\leq 0,\;\forall s$$

$$\sum_{q=1}^{Q}O_{qs}\beta_{q}-\sum_{g=1}^{G}C_{gs}\omega_{g}-\sum_{x=1}^{X}A_{xs}\mu_{x}\leq 0,\;\forall s$$

$$\alpha_{p},\lambda_{y},\omega_{g},\mu_{x},\beta_{q}\geq 0,\;\forall p,y,g,x,q$$

Please note that the optimal multipliers derived from Model (8) may not have a unique solution. As a result, the decomposition of the overall efficiency described in Eq (3) will not be unique either. Accordingly, Kao & Hwang [64] proposed a technique to identify a group of multipliers that yield the highest efficiency score for either stage 1 or stage 2, while still preserving the overall efficiency score. If we assume that the efficiency of the first stage carries more significance for the decision maker, we can estimate $\Theta_{k}^{S1}$ by solving Model (9), while optimizing $\Theta_{k}^{N*}$ through Model (8):

$$\Theta_{k}^{S1}=\mathrm{Max}\;\frac{\sum_{y=1}^{Y}L_{yk}\lambda_{y}+\sum_{g=1}^{G}C_{gk}\omega_{g}}{\sum_{p=1}^{P}I_{pk}\alpha_{p}}$$ (9)

$$S.t.\;\frac{\sum_{y=1}^{Y}L_{ys}\lambda_{y}+\sum_{g=1}^{G}C_{gs}\omega_{g}}{\sum_{p=1}^{P}I_{ps}\alpha_{p}}\leq 1,\;\forall s$$

$$\frac{\sum_{q=1}^{Q}O_{qs}\beta_{q}}{\sum_{g=1}^{G}C_{gs}\omega_{g}+\sum_{x=1}^{X}A_{xs}\mu_{x}}\leq 1,\;\forall s$$

$$\frac{\sum_{y=1}^{Y}L_{yk}\lambda_{y}+\sum_{g=1}^{G}C_{gk}\omega_{g}+\sum_{q=1}^{Q}O_{qk}\beta_{q}}{\sum_{p=1}^{P}I_{pk}\alpha_{p}+\sum_{g=1}^{G}C_{gk}\omega_{g}+\sum_{x=1}^{X}A_{xk}\mu_{x}}=\Theta_{k}^{N*}$$

$$\alpha_{p},\lambda_{y},\omega_{g},\mu_{x},\beta_{q}\geq 0,\;\forall p,y,g,x,q$$

As Model (9) is classified as a linear fractional program, through the application of conversion technique of Charnes & Cooper [63], this specific model can be considered equivalent to Model (10):

$$\Theta_{k}^{S1}=\mathrm{Max}\;\sum_{y=1}^{Y}L_{yk}\lambda_{y}+\sum_{g=1}^{G}C_{gk}\omega_{g}$$ (10)

$$S.t.\;\sum_{p=1}^{P}I_{pk}\alpha_{p}=1$$

$$\sum_{y=1}^{Y}L_{ys}\lambda_{y}+\sum_{g=1}^{G}C_{gs}\omega_{g}-\sum_{p=1}^{P}I_{ps}\alpha_{p}\leq 0,\;\forall s$$

$$\sum_{q=1}^{Q}O_{qs}\beta_{q}-\sum_{g=1}^{G}C_{gs}\omega_{g}-\sum_{x=1}^{X}A_{xs}\mu_{x}\leq 0,\;\forall s$$

$$(\sum_{y=1}^{Y}L_{yk}\lambda_{y}+\sum_{g=1}^{G}C_{gk}\omega_{g}+\sum_{q=1}^{Q}O_{qk}\beta_{q})-\Theta_{k}^{N*}(\sum_{p=1}^{P}I_{pk}\alpha_{p}+\sum_{g=1}^{G}C_{gk}\omega_{g}+\sum_{x=1}^{X}A_{xk}\mu_{x})=0$$

$$\alpha_{p},\lambda_{y},\omega_{g},\mu_{x},\beta_{q}\geq 0,\;\forall p,y,g,x,q$$

Once $\Theta_{k}^{S1*}$ is determined through the utilization of the Model (10), the efficiency score for the second stage can be acquired by employing Eq (11):

$$\Theta_{k}^{S2*}=\frac{\Theta_{k}^{N*}-\Psi_{k}^{S1*}\Theta_{k}^{S1*}}{\Psi_{k}^{S2*}}$$ (11)

It should be emphasized that the optimal weights $\Psi_{k}^{S1*}$ and $\Psi_{k}^{S2*}$ are derived from Model (8) by implementing Eqs (5) and (6). In contrast, if we consider the stage 2 to be more significant, the efficiency of stages 2 and 1 will be evaluated using a comparable approach.

## 4. The credibility-based fuzzy network DEA approach

In this particular section, we will introduce a novel FNDEA approach designed to handle imprecise and vague data. Notably, our proposed fuzzy network DEA model, adopts an additive efficiency decomposition approach and assumes that first stage holds greater significance. Consequently, we make the assumption that all the data (${\tilde{I}}_{ps}$, ${\tilde{L}}_{ys}$, ${\tilde{C}}_{gs}$, ${\tilde{A}}_{xs}$ and ${\tilde{O}}_{qs}$) involved can be considered to be approximately known, and can be represented by triangular membership functions denoted as fuzzy numbers ${\tilde{I}}_{ps}(I_{ps}^{\left(1\right)},I_{ps}^{\left(2\right)},I_{ps}^{\left(3\right)})$, ${\tilde{L}}_{ys}(L_{ys}^{\left(1\right)},L_{ys}^{\left(2\right)},L_{ys}^{\left(3\right)})$, ${\tilde{C}}_{gs}(C_{gs}^{\left(1\right)},C_{gs}^{\left(2\right)},C_{gs}^{\left(3\right)})$, ${\tilde{A}}_{xs}(A_{xs}^{\left(1\right)},A_{xs}^{\left(2\right)},A_{xs}^{\left(3\right)})$, and ${\tilde{O}}_{qs}(O_{qs}^{\left(1\right)},O_{qs}^{\left(2\right)},O_{qs}^{\left(3\right)})$ in which $I_{ps}^{\left(1\right)}<I_{ps}^{\left(2\right)}<I_{ps}^{\left(3\right)}$, $L_{ys}^{\left(1\right)}<L_{ys}^{\left(2\right)}<L_{ys}^{\left(3\right)}$, $C_{gs}^{\left(1\right)}<C_{gs}^{\left(2\right)}<C_{gs}^{\left(3\right)}$, $A_{xs}^{\left(1\right)}<A_{xs}^{\left(2\right)}<A_{xs}^{\left(3\right)}$, and $O_{qs}^{\left(1\right)}<O_{qs}^{\left(2\right)}<O_{qs}^{\left(3\right)}$. To account for the ambiguity in the available data, we can reformulate Models (8) and (10) for fuzzy observations into Models (12) and (13) correspondingly:

$${\tilde{\Theta }}_{k}^{N}=\mathrm{Max}\;ϒ$$ (12)

$$S.t.\;\sum_{y=1}^{Y}{\tilde{L}}_{yk}\lambda_{y}+\sum_{g=1}^{G}{\tilde{C}}_{gk}\omega_{g}+\sum_{q=1}^{Q}{\tilde{O}}_{qk}\beta_{q}\geq ϒ$$

$$\sum_{p=1}^{P}{\tilde{I}}_{pk}\alpha_{p}+\sum_{g=1}^{G}{\tilde{C}}_{gk}\omega_{g}+\sum_{x=1}^{X}{\tilde{A}}_{xk}\mu_{x}\leq 1$$

$$\sum_{y=1}^{Y}{\tilde{L}}_{ys}\lambda_{y}+\sum_{g=1}^{G}{\tilde{C}}_{gs}\omega_{g}-\sum_{p=1}^{P}{\tilde{I}}_{ps}\alpha_{p}\leq 0,\;\forall s$$

$$\sum_{q=1}^{Q}{\tilde{O}}_{qs}\beta_{q}-\sum_{g=1}^{G}{\tilde{C}}_{gs}\omega_{g}-\sum_{x=1}^{X}{\tilde{A}}_{xs}\mu_{x}\leq 0,\;\forall s$$

$$\alpha_{p},\lambda_{y},\omega_{g},\mu_{x},\beta_{q}\geq 0,\;\forall p,y,g,x,q$$

$${\tilde{\Theta }}_{k}^{S1}=\mathrm{Max}\;ϒ$$ (13)

$$S.t.\;\sum_{y=1}^{Y}{\tilde{L}}_{yk}\lambda_{y}+\sum_{g=1}^{G}{\tilde{C}}_{gk}\omega_{g}\geq ϒ$$

$$\sum_{p=1}^{P}{\tilde{I}}_{pk}\alpha_{p}\leq 1$$

$$\sum_{y=1}^{Y}{\tilde{L}}_{ys}\lambda_{y}+\sum_{g=1}^{G}{\tilde{C}}_{gs}\omega_{g}-\sum_{p=1}^{P}{\tilde{I}}_{ps}\alpha_{p}\leq 0,\;\forall s$$

$$\sum_{q=1}^{Q}{\tilde{O}}_{qs}\beta_{q}-\sum_{g=1}^{G}{\tilde{C}}_{gs}\omega_{g}-\sum_{x=1}^{X}{\tilde{A}}_{xs}\mu_{x}\leq 0,\;\forall s$$

$$(\sum_{y=1}^{Y}{\tilde{L}}_{yk}\lambda_{y}+\sum_{g=1}^{G}{\tilde{C}}_{gk}\omega_{g}+\sum_{q=1}^{Q}{\tilde{O}}_{qk}\beta_{q})-{\tilde{\Theta }}_{k}^{N*}(\sum_{p=1}^{P}{\tilde{I}}_{pk}\alpha_{p}+\sum_{g=1}^{G}{\tilde{C}}_{gk}\omega_{g}+\sum_{x=1}^{X}{\tilde{A}}_{xk}\mu_{x})\geq 0$$

$$\alpha_{p},\lambda_{y},\omega_{g},\mu_{x},\beta_{q}\geq 0,\;\forall p,y,g,x,q$$

As evident from the Models (12) and (13), notable modifications have occurred in both the objective function and equality constraints. However, it is important to note that these alterations do not affect the optimal solutions proposed by the models [65, 66]. Consequently, to effectively handle imprecise and ambiguous data within Models (12) and (13), we shall employ the principles of credibility theory [67] and chance-constrained programming [68]. It should be explained that the credibility (*Cr*) measure of {†} is defined on the possibility space (Ξ,*P*(Ξ),*Pos*) as the average of its possibility (*Pos*) and necessity (*Nec*) measures that is presented in Eq (14):

$$Cr\{†\}=\frac{1}{2}(Pos\{†\}+Nec\{†\})$$ (14)

Let $\tilde{\varphi }$ be a triangular fuzzy variable $\tilde{\varphi }(\varphi^{\left(1\right)},\varphi^{\left(2\right)},\varphi^{\left(3\right)})$ on the possibility space (Ξ,*P*(Ξ),*Pos*) and *η* be a crisp number. The credibility of fuzzy events $\left\{\tilde{\varphi }\leq \eta \right\}$ and $\left\{\tilde{\varphi }\geq \eta \right\}$ can be specified using Eqs (15) and (16), respectively:

$$Cr\{\tilde{\varphi }\leq \eta \}=\{\begin{array}{ll} 0, & if\;\varphi^{\left(1\right)}\geq \eta ;\\ \frac{\eta -\varphi^{\left(1\right)}}{2(\varphi^{\left(2\right)}-\varphi^{\left(1\right)})}, & if\;\varphi^{\left(1\right)}\leq \eta \leq \varphi^{\left(2\right)};\\ \frac{\eta +\varphi^{\left(3\right)}-2\varphi^{\left(2\right)}}{2(\varphi^{\left(3\right)}-\varphi^{\left(2\right)})}, & if\;\varphi^{\left(2\right)}\leq \eta \leq \varphi^{\left(3\right)};\\ 1, & if\;\varphi^{\left(3\right)}\leq \eta . \end{array}$$ (15)

$$Cr\{\tilde{\varphi }\geq \eta \}=\{\begin{array}{ll} 1, & if\;\varphi^{\left(1\right)}\geq \eta ;\\ \frac{2\varphi^{\left(2\right)}-\varphi^{\left(1\right)}-\eta }{2(\varphi^{\left(2\right)}-\varphi^{\left(1\right)})}, & if\;\varphi^{\left(1\right)}\leq \eta \leq \varphi^{\left(2\right)};\\ \frac{\varphi^{\left(3\right)}-\eta }{2(\varphi^{\left(3\right)}-\varphi^{\left(2\right)})}, & if\;\varphi^{\left(2\right)}\leq \eta \leq \varphi^{\left(3\right)};\\ 0, & if\;\varphi^{\left(3\right)}\leq \eta . \end{array}$$ (16)

In accordance with the credibility measure, the process of transforming fuzzy chance constraints into their equivalent crisp constraints at a specific confidence level *ξ* can be precisely defined utilizing Eqs (17) and (18) correspondingly [32, 65]:

$$Cr\{\tilde{\varphi }\leq \eta \}\geq \xi \Leftrightarrow \{\begin{array}{ll} (1-2\xi )\varphi^{\left(1\right)}+(2\xi )\varphi^{\left(2\right)}\leq \eta , & if\;\xi \leq 0.5;\\ (2-2\xi )\varphi^{\left(2\right)}+(2\xi -1)\varphi^{\left(3\right)}\leq \eta , & if\;\xi >0.5. \end{array}$$ (17)

$$Cr\{\tilde{\varphi }\geq \eta \}\geq \xi \Leftrightarrow \{\begin{array}{ll} (2\xi )\varphi^{\left(2\right)}+(1-2\xi )\varphi^{\left(3\right)}\geq \eta , & if\;\xi \leq 0.5;\\ (2\xi -1)\varphi^{\left(1\right)}+(2-2\xi )\varphi^{\left(2\right)}\geq \eta , & if\;\xi >0.5. \end{array}$$ (18)

In light of the credibility measure and chance-constrained programming, the Models (12) and (13) have been redefined as Models (19) and (20) correspondingly:

$${\tilde{\Theta }}_{k}^{N}=\mathrm{Max}\;ϒ$$ (19)

$$S.t.\;Cr\{\sum_{y=1}^{Y}{\tilde{L}}_{yk}\lambda_{y}+\sum_{g=1}^{G}{\tilde{C}}_{gk}\omega_{g}+\sum_{q=1}^{Q}{\tilde{O}}_{qk}\beta_{q}\geq ϒ\}\geq \xi$$

$$Cr\{\sum_{p=1}^{P}{\tilde{I}}_{pk}\alpha_{p}+\sum_{g=1}^{G}{\tilde{C}}_{gk}\omega_{g}+\sum_{x=1}^{X}{\tilde{A}}_{xk}\mu_{x}\leq 1\}\geq \xi$$

$$Cr\{\sum_{y=1}^{Y}{\tilde{L}}_{ys}\lambda_{y}+\sum_{g=1}^{G}{\tilde{C}}_{gs}\omega_{g}-\sum_{p=1}^{P}{\tilde{I}}_{ps}\alpha_{p}\leq 0\}\geq \xi ,\;\forall s$$

$$Cr\{\sum_{q=1}^{Q}{\tilde{O}}_{qs}\beta_{q}-\sum_{g=1}^{G}{\tilde{C}}_{gs}\omega_{g}-\sum_{x=1}^{X}{\tilde{A}}_{xs}\mu_{x}\leq 0\}\geq \xi ,\;\forall s$$

$$\alpha_{p},\lambda_{y},\omega_{g},\mu_{x},\beta_{q}\geq 0,\;\forall p,y,g,x,q$$

$${\tilde{\Theta }}_{k}^{S1}=\mathrm{Max}\;ϒ$$ (20)

$$S.t.\;Cr\{\sum_{y=1}^{Y}{\tilde{L}}_{yk}\lambda_{y}+\sum_{g=1}^{G}{\tilde{C}}_{gk}\omega_{g}\geq ϒ\}\geq \xi$$

$$Cr\{\sum_{p=1}^{P}{\tilde{I}}_{pk}\alpha_{p}\leq 1\}\geq \xi$$

$$Cr\{\sum_{y=1}^{Y}{\tilde{L}}_{ys}\lambda_{y}+\sum_{g=1}^{G}{\tilde{C}}_{gs}\omega_{g}-\sum_{p=1}^{P}{\tilde{I}}_{ps}\alpha_{p}\leq 0\}\geq \xi ,\;\forall s$$

$$Cr\{\sum_{q=1}^{Q}{\tilde{O}}_{qs}\beta_{q}-\sum_{g=1}^{G}{\tilde{C}}_{gs}\omega_{g}-\sum_{x=1}^{X}{\tilde{A}}_{xs}\mu_{x}\leq 0\}\geq \xi ,\;\forall s$$

$$Cr\{(\sum_{y=1}^{Y}{\tilde{L}}_{yk}\lambda_{y}+\sum_{g=1}^{G}{\tilde{C}}_{gk}\omega_{g}+\sum_{q=1}^{Q}{\tilde{O}}_{qk}\beta_{q})-{\tilde{\Theta }}_{k}^{N*}(\sum_{p=1}^{P}{\tilde{I}}_{pk}\alpha_{p}+\sum_{g=1}^{G}{\tilde{C}}_{gk}\omega_{g}+\sum_{x=1}^{X}{\tilde{A}}_{xk}\mu_{x})\geq 0\}\geq \xi$$

$$\alpha_{p},\lambda_{y},\omega_{g},\mu_{x},\beta_{q}\geq 0,\;\forall p,y,g,x,q$$

Based on Eqs (17) and (18), if the confidence levels are above or below 0.5, the crisp counterpart of fuzzy chance constraints would differ. It is important to mention that by utilizing a binary variable Ω and a significantly large value Γ, one can streamline the process of linearization of incompatible constraints and seamlessly incorporate fuzzy NDEA models for *ξ*≤0.5 and *ξ*>0.5. As a result, we introduce the credibility-based fuzzy network DEA approach for determining the overall efficiency score of a DMU_*k*_ considering fuzzy data. This model is denoted as Model (21):

$$\Theta_{k(\xi )}^{N}=\mathrm{Max}\;ϒ$$ (21)

$$\begin{array}{l} S.t.\;\sum_{y=1}^{Y}((2\xi )L_{yk}^{\left(2\right)}+(1-2\xi )L_{yk}^{\left(3\right)})\lambda_{y}+\sum_{g=1}^{G}((2\xi )C_{gk}^{\left(2\right)}+(1-2\xi )C_{gk}^{\left(3\right)})\omega_{g}\\ \;+\sum_{q=1}^{Q}((2\xi )O_{qk}^{\left(2\right)}+(1-2\xi )O_{qk}^{\left(3\right)})\beta_{q}\geq ϒ-\Gamma \Omega \end{array}$$

$$\begin{array}{l} \;\sum_{y=1}^{Y}((2\xi -1)L_{yk}^{\left(1\right)}+(2-2\xi )L_{yk}^{\left(2\right)})\lambda_{y}+\sum_{g=1}^{G}((2\xi -1)C_{gk}^{\left(1\right)}+(2-2\xi )C_{gk}^{\left(2\right)})\omega_{g}\\ +\sum_{q=1}^{Q}((2\xi -1)O_{qk}^{\left(1\right)}+(2-2\xi )O_{qk}^{\left(2\right)})\beta_{q}\geq ϒ-\Gamma (1-\Omega ) \end{array}$$

$$\begin{array}{l} \;\sum_{p=1}^{P}((1-2\xi )I_{pk}^{\left(1\right)}+(2\xi )I_{pk}^{\left(2\right)})\alpha_{p}+\sum_{g=1}^{G}((1-2\xi )C_{gk}^{\left(1\right)}+(2\xi )C_{gk}^{\left(2\right)})\omega_{g}\\ +\sum_{x=1}^{X}((1-2\xi )A_{xk}^{\left(1\right)}+(2\xi )A_{xk}^{\left(2\right)})\mu_{x}\leq 1+\Gamma \Omega \end{array}$$

$$\begin{array}{l} \;\sum_{p=1}^{P}((2-2\xi )I_{pk}^{\left(2\right)}+(2\xi -1)I_{pk}^{\left(3\right)})\alpha_{p}+\sum_{g=1}^{G}((2-2\xi )C_{gk}^{\left(2\right)}+(2\xi -1)C_{gk}^{\left(3\right)})\omega_{g}\\ +\sum_{x=1}^{X}((2-2\xi )A_{xk}^{\left(2\right)}+(2\xi -1)A_{xk}^{\left(3\right)})\mu_{x}\leq 1+\Gamma (1-\Omega ) \end{array}$$

$$\begin{array}{l} \;\sum_{y=1}^{Y}((1-2\xi )L_{ys}^{\left(1\right)}+(2\xi )L_{ys}^{\left(2\right)})\lambda_{y}+\sum_{g=1}^{G}((1-2\xi )C_{gk}^{\left(1\right)}+(2\xi )C_{gk}^{\left(2\right)})\omega_{g}\\ -\sum_{p=1}^{P}((2\xi )I_{ps}^{\left(2\right)}+(1-2\xi )I_{ps}^{\left(3\right)})\alpha_{p}\leq \Gamma \Omega ,\;\forall s \end{array}$$

$$\begin{array}{l} \;\sum_{y=1}^{Y}((2-2\xi )L_{ys}^{\left(2\right)}+(2\xi -1)L_{ys}^{\left(3\right)})\lambda_{y}+\sum_{g=1}^{G}((2-2\xi )C_{gk}^{\left(2\right)}+(2\xi -1)C_{gk}^{\left(3\right)})\omega_{g}\\ -\sum_{p=1}^{P}((2\xi -1)I_{ps}^{\left(1\right)}+(2-2\xi )I_{ps}^{\left(2\right)})\alpha_{p}\leq \Gamma (1-\Omega ),\;\forall s \end{array}$$

$$\begin{array}{l} \;\sum_{q=1}^{Q}((1-2\xi )O_{qs}^{\left(1\right)}+(2\xi )O_{qs}^{\left(2\right)})\beta_{q}-\sum_{g=1}^{G}((2\xi )C_{gs}^{\left(2\right)}+(1-2\xi )C_{gs}^{\left(3\right)})\omega_{g}\\ -\sum_{x=1}^{X}((2\xi )A_{xs}^{\left(2\right)}+(1-2\xi )A_{xs}^{\left(3\right)})\mu_{x}\leq \Gamma \Omega ,\;\forall s \end{array}$$

$$\begin{array}{l} \;\sum_{q=1}^{Q}((2-2\xi )O_{qs}^{\left(2\right)}+(2\xi -1)O_{qs}^{\left(3\right)})\beta_{q}-\sum_{g=1}^{G}((2\xi -1)C_{gs}^{\left(1\right)}+(2-2\xi )C_{gs}^{\left(2\right)})\omega_{g}\\ -\sum_{x=1}^{X}((2\xi -1)A_{xs}^{\left(1\right)}+(2-2\xi )A_{xs}^{\left(2\right)})\mu_{x}\leq \Gamma (1-\Omega ),\;\forall s \end{array}$$

ξ≤0.5+ΓΩ

ξ>0.5−Γ(1−Ω)

Ω∈{0,1}

$$\alpha_{p},\lambda_{y},\omega_{g},\mu_{x},\beta_{q}\geq 0,\;\forall p,y,g,x,q$$

Next, we proceed to estimate the efficiency score of the first stage considering the introduction of fuzzy data. This estimation is achieved by solving Model (22), with the values of $\Theta_{k(\xi )}^{N*}$ being obtained from Model (21):

$$\Theta_{k(\xi )}^{S1}=\mathrm{Max}\;ϒ$$ (22)

$$S.t.\;\sum_{y=1}^{Y}((2\xi )L_{yk}^{\left(2\right)}+(1-2\xi )L_{yk}^{\left(3\right)})\lambda_{y}+\sum_{g=1}^{G}((2\xi )C_{gk}^{\left(2\right)}+(1-2\xi )C_{gk}^{\left(3\right)})\omega_{g}\geq ϒ-\Gamma \Omega$$

$$\sum_{y=1}^{Y}((2\xi -1)L_{yk}^{\left(1\right)}+(2-2\xi )L_{yk}^{\left(2\right)})\lambda_{y}+\sum_{g=1}^{G}((2\xi -1)C_{gk}^{\left(1\right)}+(2-2\xi )C_{gk}^{\left(2\right)})\omega_{g}\geq ϒ-\Gamma (1-\Omega )$$

$$\sum_{p=1}^{P}((1-2\xi )I_{pk}^{\left(1\right)}+(2\xi )I_{pk}^{\left(2\right)})\alpha_{p}\leq 1+\Gamma \Omega$$

$$\sum_{p=1}^{P}((2-2\xi )I_{pk}^{\left(2\right)}+(2\xi -1)I_{pk}^{\left(3\right)})\alpha_{p}\leq 1+\Gamma (1-\Omega )$$

$$\begin{array}{l} \;\sum_{y=1}^{Y}((1-2\xi )L_{ys}^{\left(1\right)}+(2\xi )L_{ys}^{\left(2\right)})\lambda_{y}+\sum_{g=1}^{G}((1-2\xi )C_{gk}^{\left(1\right)}+(2\xi )C_{gk}^{\left(2\right)})\omega_{g}\\ -\sum_{p=1}^{P}((2\xi )I_{ps}^{\left(2\right)}+(1-2\xi )I_{ps}^{\left(3\right)})\alpha_{p}\leq \Gamma \Omega ,\;\forall s \end{array}$$

$$\begin{array}{l} \;\sum_{y=1}^{Y}((2-2\xi )L_{ys}^{\left(2\right)}+(2\xi -1)L_{ys}^{\left(3\right)})\lambda_{y}+\sum_{g=1}^{G}((2-2\xi )C_{gk}^{\left(2\right)}+(2\xi -1)C_{gk}^{\left(3\right)})\omega_{g}\\ -\sum_{p=1}^{P}((2\xi -1)I_{ps}^{\left(1\right)}+(2-2\xi )I_{ps}^{\left(2\right)})\alpha_{p}\leq \Gamma (1-\Omega ),\;\forall s \end{array}$$

$$\begin{array}{l} \;\sum_{q=1}^{Q}((1-2\xi )O_{qs}^{\left(1\right)}+(2\xi )O_{qs}^{\left(2\right)})\beta_{q}-\sum_{g=1}^{G}((2\xi )C_{gs}^{\left(2\right)}+(1-2\xi )C_{gs}^{\left(3\right)})\omega_{g}\\ -\sum_{x=1}^{X}((2\xi )A_{xs}^{\left(2\right)}+(1-2\xi )A_{xs}^{\left(3\right)})\mu_{x}\leq \Gamma \Omega ,\;\forall s \end{array}$$

$$\begin{array}{l} \;\sum_{q=1}^{Q}((2-2\xi )O_{qs}^{\left(2\right)}+(2\xi -1)O_{qs}^{\left(3\right)})\beta_{q}-\sum_{g=1}^{G}((2\xi -1)C_{gs}^{\left(1\right)}+(2-2\xi )C_{gs}^{\left(2\right)})\omega_{g}\\ -\sum_{x=1}^{X}((2\xi -1)A_{xs}^{\left(1\right)}+(2-2\xi )A_{xs}^{\left(2\right)})\mu_{x}\leq \Gamma (1-\Omega ),\;\forall s \end{array}$$

1


$$\begin{array}{l} \;(\sum_{y=1}^{Y}((2\xi )L_{yk}^{\left(2\right)}+(1-2\xi )L_{yk}^{\left(3\right)})\lambda_{y}+\sum_{g=1}^{G}((2\xi )C_{gk}^{\left(2\right)}+(1-2\xi )C_{gk}^{\left(3\right)})\omega_{g}\\ +\sum_{q=1}^{Q}((2\xi )O_{qk}^{\left(2\right)}+(1-2\xi )O_{qk}^{\left(3\right)})\beta_{q})-\Theta_{k(\xi )}^{N*}(\sum_{p=1}^{P}((1-2\xi )I_{pk}^{\left(1\right)}+(2\xi )I_{pk}^{\left(2\right)})\alpha_{p}\\ +\sum_{g=1}^{G}((1-2\xi )C_{gk}^{\left(1\right)}+(2\xi )C_{gk}^{\left(2\right)})\omega_{g}+\sum_{x=1}^{X}((1-2\xi )A_{xk}^{\left(1\right)}+(2\xi )A_{xk}^{\left(2\right)})\mu_{x})\geq -\Gamma \Omega \end{array}$$

$$\begin{array}{l} \;(\sum_{y=1}^{Y}((2\xi -1)L_{yk}^{\left(1\right)}+(2-2\xi )L_{yk}^{\left(2\right)})\lambda_{y}+\sum_{g=1}^{G}((2\xi -1)C_{gk}^{\left(1\right)}+(2-2\xi )C_{gk}^{\left(2\right)})\omega_{g}\\ +\sum_{q=1}^{Q}((2\xi -1)O_{qk}^{\left(1\right)}+(2-2\xi )O_{qk}^{\left(2\right)})\beta_{q})-\Theta_{k(\xi )}^{N*}(((\sum_{p=1}^{P}((2-2\xi )I_{pk}^{\left(2\right)}+(2\xi -1)I_{pk}^{\left(3\right)})\alpha_{p}\\ +\sum_{g=1}^{G}((2-2\xi )C_{gk}^{\left(2\right)}+(2\xi -1)C_{gk}^{\left(3\right)})\omega_{g}+\sum_{x=1}^{X}((2-2\xi )A_{xk}^{\left(2\right)}+(2\xi -1)A_{xk}^{\left(3\right)})\mu_{x})\geq -\Gamma (1-\Omega ) \end{array}$$

ξ≤0.5+ΓΩ

ξ>0.5−Γ(1−Ω)

Ω∈{0,1}

$$\alpha_{p},\lambda_{y},\omega_{g},\mu_{x},\beta_{q}\geq 0,\;\forall p,y,g,x,q$$

Ultimately, upon deriving the value of $\Psi_{k(\xi )}^{S1*}$ from Model (22), we can proceed to assess the efficiency score of second stage by employing Eq (23):

$$\Theta_{k(\xi )}^{S2*}=\frac{\Theta_{k(\xi )}^{N*}-\Psi_{k(\xi )}^{S1*}\Theta_{k(\xi )}^{S1*}}{\Psi_{k(\xi )}^{S2*}}$$ (23)

It is important to acknowledge that the efficiency scores derived from the proposed credibility-based fuzzy network DEA approach can surpass a value of one.

## 5. The proposed Malmquist productivity index

The Malmquist productivity index, is a productivity measurement tool that evaluates the change in productivity over time. It takes into account the efficiency change and technological change components to analyze productivity growth or decline [21]. The integration of MPI and DEA involves using the efficiency scores obtained from DEA as inputs to calculate the Malmquist productivity index. This allows for a more comprehensive assessment of productivity change by considering both efficiency improvements and technological advancements. By combining the two approaches, the integration of MPI and DEA provides a more robust analysis of productivity change over time. It helps identify the sources of productivity growth or decline, determine the contribution of efficiency improvements versus technological progress, and compare the performance of different DMUs over multiple periods.

In this section, we introduce an improved framework for calculating the Malmquist productivity index specifically designed for two-stage network systems. Our approach takes into account the presence of fuzzy data, which enables a more comprehensive and accurate analysis. By addressing the limitations of traditional methodologies, we enhance the evaluation and comparison of productivity change in complex network systems. Moreover, by considering fuzzy data, our methodology enables a more nuanced and complete understanding of performance variations within network systems. This enhanced understanding allows organizations to make more informed decisions and formulate effective strategies to improve productivity. The calculation of the Malmquist productivity index for two-stage network systems under fuzzy data involves a multi-step process. Here are the key steps:

**Step 1:** Determine the specific time period for which you want to calculate the Malmquist productivity index. It could be a year, a quarter, or any other defined time frame.

**Step 2:** Collect the essential data regarding inputs and outputs for the decision-making units that are intended for analysis.

**Step 3:** Apply the proposed credibility-based fuzzy network data envelopment analysis approach to calculate the efficiency scores for the two-stage DMUs in the given time period.

**Step 4:** Calculate the components of the MPI for each DMU. The MPI consists of two components: efficiency change and technological change.

**Efficiency Change:** Measure the change in efficiency for each DMU between time periods *t* and *t*+1. This is done by comparing the efficiency scores obtained from DEA for the two time periods. Notably, in order to derive the overall efficiency score of a DMU_*k*_ for two time periods, the proposed credibility-based fuzzy network DEA approach is executed on a dataset that pertains to time periods *t* and *t*+1. Our method utilizes Models (24) and (25) to evaluate the overall efficiency score of the DMU_*k*_ for time periods *t* and *t*+1, respectively:

$$\Theta_{k(\xi )}^{N(t)}(I_{k}^{t},L_{k}^{t},C_{k}^{t},A_{k}^{t},O_{k}^{t})=\mathrm{Max}\;ϒ$$ (24)

$$\begin{array}{l} S.t.\;\sum_{y=1}^{Y}((2\xi )L_{yk}^{(2)t}+(1-2\xi )L_{yk}^{(3)t})\lambda_{y}+\sum_{g=1}^{G}((2\xi )C_{gk}^{(2)t}+(1-2\xi )C_{gk}^{(3)t})\omega_{g}\\ \;+\sum_{q=1}^{Q}((2\xi )O_{qk}^{(2)t}+(1-2\xi )O_{qk}^{(3)t})\beta_{q}\geq ϒ-\Gamma \Omega \end{array}$$

$$\begin{array}{l} \;\sum_{y=1}^{Y}((2\xi -1)L_{yk}^{(1)t}+(2-2\xi )L_{yk}^{(2)t})\lambda_{y}+\sum_{g=1}^{G}((2\xi -1)C_{gk}^{(1)t}+(2-2\xi )C_{gk}^{(2)t})\omega_{g}\\ +\sum_{q=1}^{Q}((2\xi -1)O_{qk}^{(1)t}+(2-2\xi )O_{qk}^{(2)t})\beta_{q}\geq ϒ-\Gamma (1-\Omega ) \end{array}$$

$$\begin{array}{l} \;\sum_{p=1}^{P}((1-2\xi )I_{pk}^{(1)t}+(2\xi )I_{pk}^{(2)t})\alpha_{p}+\sum_{g=1}^{G}((1-2\xi )C_{gk}^{(1)t}+(2\xi )C_{gk}^{(2)t})\omega_{g}\\ +\sum_{x=1}^{X}((1-2\xi )A_{xk}^{(1)t}+(2\xi )A_{xk}^{(2)t})\mu_{x}\leq 1+\Gamma \Omega \end{array}$$

$$\begin{array}{l} \;\sum_{p=1}^{P}((2-2\xi )I_{pk}^{(2)t}+(2\xi -1)I_{pk}^{(3)t})\alpha_{p}+\sum_{g=1}^{G}((2-2\xi )C_{gk}^{(2)t}+(2\xi -1)C_{gk}^{(3)t})\omega_{g}\\ +\sum_{x=1}^{X}((2-2\xi )A_{xk}^{(2)t}+(2\xi -1)A_{xk}^{(3)t})\mu_{x}\leq 1+\Gamma (1-\Omega ) \end{array}$$

$$\begin{array}{l} \;\sum_{y=1}^{Y}((1-2\xi )L_{ys}^{(1)t}+(2\xi )L_{ys}^{(2)t})\lambda_{y}+\sum_{g=1}^{G}((1-2\xi )C_{gk}^{(1)t}+(2\xi )C_{gk}^{(2)t})\omega_{g}\\ -\sum_{p=1}^{P}((2\xi )I_{ps}^{(2)t}+(1-2\xi )I_{ps}^{(3)t})\alpha_{p}\leq \Gamma \Omega ,\;\forall s \end{array}$$

$$\begin{array}{l} \;\sum_{y=1}^{Y}((2-2\xi )L_{ys}^{(2)t}+(2\xi -1)L_{ys}^{(3)t})\lambda_{y}+\sum_{g=1}^{G}((2-2\xi )C_{gk}^{(2)t}+(2\xi -1)C_{gk}^{(3)t})\omega_{g}\\ -\sum_{p=1}^{P}((2\xi -1)I_{ps}^{(1)t}+(2-2\xi )I_{ps}^{(2)t})\alpha_{p}\leq \Gamma (1-\Omega ),\;\forall s \end{array}$$

$$\begin{array}{l} \;\sum_{q=1}^{Q}((1-2\xi )O_{qs}^{(1)t}+(2\xi )O_{qs}^{(2)t})\beta_{q}-\sum_{g=1}^{G}((2\xi )C_{gs}^{(2)t}+(1-2\xi )C_{gs}^{(3)t})\omega_{g}\\ -\sum_{x=1}^{X}((2\xi )A_{xs}^{(2)t}+(1-2\xi )A_{xs}^{(3)t})\mu_{x}\leq \Gamma \Omega ,\;\forall s \end{array}$$

$$\begin{array}{l} \;\sum_{q=1}^{Q}((2-2\xi )O_{qs}^{(2)t}+(2\xi -1)O_{qs}^{(3)t})\beta_{q}-\sum_{g=1}^{G}((2\xi -1)C_{gs}^{(1)t}+(2-2\xi )C_{gs}^{(2)t})\omega_{g}\\ -\sum_{x=1}^{X}((2\xi -1)A_{xs}^{(1)t}+(2-2\xi )A_{xs}^{(2)t})\mu_{x}\leq \Gamma (1-\Omega ),\;\forall s \end{array}$$

ξ≤0.5+ΓΩ

ξ>0.5−Γ(1−Ω)

Ω∈{0,1}

$$\alpha_{p},\lambda_{y},\omega_{g},\mu_{x},\beta_{q}\geq 0,\;\forall p,y,g,x,q$$

$$\Theta_{k(\xi )}^{N(t+1)}(I_{k}^{t+1},L_{k}^{t+1},C_{k}^{t+1},A_{k}^{t+1},O_{k}^{t+1})=\mathrm{Max}\;ϒ$$ (25)

$$\begin{array}{l} S.t.\;\sum_{y=1}^{Y}((2\xi )L_{yk}^{(2)t+1}+(1-2\xi )L_{yk}^{(3)t+1})\lambda_{y}+\sum_{g=1}^{G}((2\xi )C_{gk}^{(2)t+1}+(1-2\xi )C_{gk}^{(3)t+1})\omega_{g}\\ \;+\sum_{q=1}^{Q}((2\xi )O_{qk}^{(2)t+1}+(1-2\xi )O_{qk}^{(3)t+1})\beta_{q}\geq ϒ-\Gamma \Omega \end{array}$$

$$\begin{array}{l} \;\sum_{y=1}^{Y}((2\xi -1)L_{yk}^{(1)t+1}+(2-2\xi )L_{yk}^{(2)t+1})\lambda_{y}+\sum_{g=1}^{G}((2\xi -1)C_{gk}^{(1)t+1}+(2-2\xi )C_{gk}^{(2)t+1})\omega_{g}\\ +\sum_{q=1}^{Q}((2\xi -1)O_{qk}^{(1)t+1}+(2-2\xi )O_{qk}^{(2)t+1})\beta_{q}\geq ϒ-\Gamma (1-\Omega ) \end{array}$$

$$\begin{array}{l} \;\sum_{p=1}^{P}((1-2\xi )I_{pk}^{(1)t+1}+(2\xi )I_{pk}^{(2)t+1})\alpha_{p}+\sum_{g=1}^{G}((1-2\xi )C_{gk}^{(1)t+1}+(2\xi )C_{gk}^{(2)t+1})\omega_{g}\\ +\sum_{x=1}^{X}((1-2\xi )A_{xk}^{(1)t+1}+(2\xi )A_{xk}^{(2)t+1})\mu_{x}\leq 1+\Gamma \Omega \end{array}$$

$$\begin{array}{l} \;\sum_{p=1}^{P}((2-2\xi )I_{pk}^{(2)t+1}+(2\xi -1)I_{pk}^{(3)t+1})\alpha_{p}+\sum_{g=1}^{G}((2-2\xi )C_{gk}^{(2)t+1}+(2\xi -1)C_{gk}^{(3)t+1})\omega_{g}\\ +\sum_{x=1}^{X}((2-2\xi )A_{xk}^{(2)t+1}+(2\xi -1)A_{xk}^{(3)t+1})\mu_{x}\leq 1+\Gamma (1-\Omega ) \end{array}$$

$$\begin{array}{l} \;\sum_{y=1}^{Y}((1-2\xi )L_{ys}^{(1)t+1}+(2\xi )L_{ys}^{(2)t+1})\lambda_{y}+\sum_{g=1}^{G}((1-2\xi )C_{gk}^{(1)t+1}+(2\xi )C_{gk}^{(2)t+1})\omega_{g}\\ -\sum_{p=1}^{P}((2\xi )I_{ps}^{(2)t+1}+(1-2\xi )I_{ps}^{(3)t+1})\alpha_{p}\leq \Gamma \Omega ,\;\forall s \end{array}$$

$$\begin{array}{l} \;\sum_{y=1}^{Y}((2-2\xi )L_{ys}^{(2)t+1}+(2\xi -1)L_{ys}^{(3)t+1})\lambda_{y}+\sum_{g=1}^{G}((2-2\xi )C_{gk}^{(2)t+1}+(2\xi -1)C_{gk}^{(3)t+1})\omega_{g}\\ -\sum_{p=1}^{P}((2\xi -1)I_{ps}^{(1)t+1}+(2-2\xi )I_{ps}^{(2)t+1})\alpha_{p}\leq \Gamma (1-\Omega ),\;\forall s \end{array}$$

$$\begin{array}{l} \;\sum_{q=1}^{Q}((1-2\xi )O_{qs}^{(1)t+1}+(2\xi )O_{qs}^{(2)t+1})\beta_{q}-\sum_{g=1}^{G}((2\xi )C_{gs}^{(2)t+1}+(1-2\xi )C_{gs}^{(3)t+1})\omega_{g}\\ -\sum_{x=1}^{X}((2\xi )A_{xs}^{(2)t+1}+(1-2\xi )A_{xs}^{(3)t+1})\mu_{x}\leq \Gamma \Omega ,\;\forall s \end{array}$$

$$\begin{array}{l} \;\sum_{q=1}^{Q}((2-2\xi )O_{qs}^{(2)t+1}+(2\xi -1)O_{qs}^{(3)t+1})\beta_{q}-\sum_{g=1}^{G}((2\xi -1)C_{gs}^{(1)t+1}+(2-2\xi )C_{gs}^{(2)t+1})\omega_{g}\\ -\sum_{x=1}^{X}((2\xi -1)A_{xs}^{(1)t+1}+(2-2\xi )A_{xs}^{(2)t+1})\mu_{x}\leq \Gamma (1-\Omega ),\;\forall s \end{array}$$

ξ≤0.5+ΓΩ

ξ>0.5−Γ(1−Ω)

Ω∈{0,1}

$$\alpha_{p},\lambda_{y},\omega_{g},\mu_{x},\beta_{q}\geq 0,\;\forall p,y,g,x,q$$

**Technological Change:** Assess the technological progress between time periods *t* and *t*+1. This is determined by comparing the production frontiers of the two time periods. Accordingly, in order to derive the technological change of a DMU_*k*_ for two time periods *t* and *t*+1, Models (26) and (27) are applied as follows:

$$\Theta_{k(\xi )}^{N(t)}(I_{k}^{t+1},L_{k}^{t+1},C_{k}^{t+1},A_{k}^{t+1},O_{k}^{t+1})=\mathrm{Max}\;ϒ$$ (26)

$$\begin{array}{l} S.t.\;\sum_{y=1}^{Y}((2\xi )L_{yk}^{(2)t+1}+(1-2\xi )L_{yk}^{(3)t+1})\lambda_{y}+\sum_{g=1}^{G}((2\xi )C_{gk}^{(2)t+1}+(1-2\xi )C_{gk}^{(3)t+1})\omega_{g}\\ \;+\sum_{q=1}^{Q}((2\xi )O_{qk}^{(2)t+1}+(1-2\xi )O_{qk}^{(3)t+1})\beta_{q}\geq ϒ-\Gamma \Omega \end{array}$$

$$\begin{array}{l} \;\sum_{y=1}^{Y}((2\xi -1)L_{yk}^{(1)t+1}+(2-2\xi )L_{yk}^{(2)t+1})\lambda_{y}+\sum_{g=1}^{G}((2\xi -1)C_{gk}^{(1)t+1}+(2-2\xi )C_{gk}^{(2)t+1})\omega_{g}\\ +\sum_{q=1}^{Q}((2\xi -1)O_{qk}^{(1)t+1}+(2-2\xi )O_{qk}^{(2)t+1})\beta_{q}\geq ϒ-\Gamma (1-\Omega ) \end{array}$$

$$\begin{array}{l} \;\sum_{p=1}^{P}((1-2\xi )I_{pk}^{(1)t+1}+(2\xi )I_{pk}^{(2)t+1})\alpha_{p}+\sum_{g=1}^{G}((1-2\xi )C_{gk}^{(1)t+1}+(2\xi )C_{gk}^{(2)t+1})\omega_{g}\\ +\sum_{x=1}^{X}((1-2\xi )A_{xk}^{(1)t+1}+(2\xi )A_{xk}^{(2)t+1})\mu_{x}\leq 1+\Gamma \Omega \end{array}$$

$$\begin{array}{l} \;\sum_{p=1}^{P}((2-2\xi )I_{pk}^{(2)t+1}+(2\xi -1)I_{pk}^{(3)t+1})\alpha_{p}+\sum_{g=1}^{G}((2-2\xi )C_{gk}^{(2)t+1}+(2\xi -1)C_{gk}^{(3)t+1})\omega_{g}\\ +\sum_{x=1}^{X}((2-2\xi )A_{xk}^{(2)t+1}+(2\xi -1)A_{xk}^{(3)t+1})\mu_{x}\leq 1+\Gamma (1-\Omega ) \end{array}$$

$$\begin{array}{l} \;\sum_{y=1}^{Y}((1-2\xi )L_{ys}^{(1)t}+(2\xi )L_{ys}^{(2)t})\lambda_{y}+\sum_{g=1}^{G}((1-2\xi )C_{gk}^{(1)t}+(2\xi )C_{gk}^{(2)t})\omega_{g}\\ -\sum_{p=1}^{P}((2\xi )I_{ps}^{(2)t}+(1-2\xi )I_{ps}^{(3)t})\alpha_{p}\leq \Gamma \Omega ,\;\forall s \end{array}$$

$$\begin{array}{l} \;\sum_{y=1}^{Y}((2-2\xi )L_{ys}^{(2)t}+(2\xi -1)L_{ys}^{(3)t})\lambda_{y}+\sum_{g=1}^{G}((2-2\xi )C_{gk}^{(2)t}+(2\xi -1)C_{gk}^{(3)t})\omega_{g}\\ -\sum_{p=1}^{P}((2\xi -1)I_{ps}^{(1)t}+(2-2\xi )I_{ps}^{(2)t})\alpha_{p}\leq \Gamma (1-\Omega ),\;\forall s \end{array}$$

$$\begin{array}{l} \;\sum_{q=1}^{Q}((1-2\xi )O_{qs}^{(1)t}+(2\xi )O_{qs}^{(2)t})\beta_{q}-\sum_{g=1}^{G}((2\xi )C_{gs}^{(2)t}+(1-2\xi )C_{gs}^{(3)t})\omega_{g}\\ -\sum_{x=1}^{X}((2\xi )A_{xs}^{(2)t}+(1-2\xi )A_{xs}^{(3)t})\mu_{x}\leq \Gamma \Omega ,\;\forall s \end{array}$$

$$\begin{array}{l} \;\sum_{q=1}^{Q}((2-2\xi )O_{qs}^{(2)t}+(2\xi -1)O_{qs}^{(3)t})\beta_{q}-\sum_{g=1}^{G}((2\xi -1)C_{gs}^{(1)t}+(2-2\xi )C_{gs}^{(2)t})\omega_{g}\\ -\sum_{x=1}^{X}((2\xi -1)A_{xs}^{(1)t}+(2-2\xi )A_{xs}^{(2)t})\mu_{x}\leq \Gamma (1-\Omega ),\;\forall s \end{array}$$

ξ≤0.5+ΓΩ

ξ>0.5−Γ(1−Ω)

Ω∈{0,1}

$$\alpha_{p},\lambda_{y},\omega_{g},\mu_{x},\beta_{q}\geq 0,\;\forall p,y,g,x,q$$

$$\Theta_{k(\xi )}^{N(t+1)}(I_{k}^{t},L_{k}^{t},C_{k}^{t},A_{k}^{t},O_{k}^{t})=\mathrm{Max}\;ϒ$$ (27)

$$\begin{array}{l} S.t.\;\sum_{y=1}^{Y}((2\xi )L_{yk}^{(2)t}+(1-2\xi )L_{yk}^{(3)t})\lambda_{y}+\sum_{g=1}^{G}((2\xi )C_{gk}^{(2)t}+(1-2\xi )C_{gk}^{(3)t})\omega_{g}\\ \;+\sum_{q=1}^{Q}((2\xi )O_{qk}^{(2)t}+(1-2\xi )O_{qk}^{(3)t})\beta_{q}\geq ϒ-\Gamma \Omega \end{array}$$

$$\begin{array}{l} \;\sum_{y=1}^{Y}((2\xi -1)L_{yk}^{(1)t}+(2-2\xi )L_{yk}^{(2)t})\lambda_{y}+\sum_{g=1}^{G}((2\xi -1)C_{gk}^{(1)t}+(2-2\xi )C_{gk}^{(2)t})\omega_{g}\\ +\sum_{q=1}^{Q}((2\xi -1)O_{qk}^{(1)t}+(2-2\xi )O_{qk}^{(2)t})\beta_{q}\geq ϒ-\Gamma (1-\Omega ) \end{array}$$

$$\begin{array}{l} \;\sum_{p=1}^{P}((1-2\xi )I_{pk}^{(1)t}+(2\xi )I_{pk}^{(2)t})\alpha_{p}+\sum_{g=1}^{G}((1-2\xi )C_{gk}^{(1)t}+(2\xi )C_{gk}^{(2)t})\omega_{g}\\ +\sum_{x=1}^{X}((1-2\xi )A_{xk}^{(1)t}+(2\xi )A_{xk}^{(2)t})\mu_{x}\leq 1+\Gamma \Omega \end{array}$$

$$\begin{array}{l} \;\sum_{p=1}^{P}((2-2\xi )I_{pk}^{(2)t}+(2\xi -1)I_{pk}^{(3)t})\alpha_{p}+\sum_{g=1}^{G}((2-2\xi )C_{gk}^{(2)t}+(2\xi -1)C_{gk}^{(3)t})\omega_{g}\\ +\sum_{x=1}^{X}((2-2\xi )A_{xk}^{(2)t}+(2\xi -1)A_{xk}^{(3)t})\mu_{x}\leq 1+\Gamma (1-\Omega ) \end{array}$$

$$\begin{array}{l} \;\sum_{y=1}^{Y}((1-2\xi )L_{ys}^{(1)t+1}+(2\xi )L_{ys}^{(2)t+1})\lambda_{y}+\sum_{g=1}^{G}((1-2\xi )C_{gk}^{(1)t+1}+(2\xi )C_{gk}^{(2)t+1})\omega_{g}\\ -\sum_{p=1}^{P}((2\xi )I_{ps}^{(2)t+1}+(1-2\xi )I_{ps}^{(3)t+1})\alpha_{p}\leq \Gamma \Omega ,\;\forall s \end{array}$$

$$\begin{array}{l} \;\sum_{y=1}^{Y}((2-2\xi )L_{ys}^{(2)t+1}+(2\xi -1)L_{ys}^{(3)t+1})\lambda_{y}+\sum_{g=1}^{G}((2-2\xi )C_{gk}^{(2)t+1}+(2\xi -1)C_{gk}^{(3)t+1})\omega_{g}\\ -\sum_{p=1}^{P}((2\xi -1)I_{ps}^{(1)t+1}+(2-2\xi )I_{ps}^{(2)t+1})\alpha_{p}\leq \Gamma (1-\Omega ),\;\forall s \end{array}$$

$$\begin{array}{l} \;\sum_{q=1}^{Q}((1-2\xi )O_{qs}^{(1)t+1}+(2\xi )O_{qs}^{(2)t+1})\beta_{q}-\sum_{g=1}^{G}((2\xi )C_{gs}^{(2)t+1}+(1-2\xi )C_{gs}^{(3)t+1})\omega_{g}\\ -\sum_{x=1}^{X}((2\xi )A_{xs}^{(2)t+1}+(1-2\xi )A_{xs}^{(3)t+1})\mu_{x}\leq \Gamma \Omega ,\;\forall s \end{array}$$

$$\begin{array}{l} \;\sum_{q=1}^{Q}((2-2\xi )O_{qs}^{(2)t+1}+(2\xi -1)O_{qs}^{(3)t+1})\beta_{q}-\sum_{g=1}^{G}((2\xi -1)C_{gs}^{(1)t+1}+(2-2\xi )C_{gs}^{(2)t+1})\omega_{g}\\ -\sum_{x=1}^{X}((2\xi -1)A_{xs}^{(1)t+1}+(2-2\xi )A_{xs}^{(2)t+1})\mu_{x}\leq \Gamma (1-\Omega ),\;\forall s \end{array}$$

ξ≤0.5+ΓΩ

ξ>0.5−Γ(1−Ω)

Ω∈{0,1}

$$\alpha_{p},\lambda_{y},\omega_{g},\mu_{x},\beta_{q}\geq 0,\;\forall p,y,g,x,q$$

In the same manner, the mentioned results are calculated for the first and second stages, and the corresponding models and relationships are available in the S1 Appendix.

**Step 5:** Combine the efficiency change and technological change components to compute the credibility-based fuzzy network Malmquist productivity index (CFNMPI) for each DMU. The CFNMPI is calculated as the geometric mean of the efficiency change and technological change.


$${\mathrm{CFNMPI}}_{k(\xi )}^{N}=\sqrt{\frac{\Theta_{k(\xi )}^{N(t)}(I_{k}^{t+1},L_{k}^{t+1},C_{k}^{t+1},A_{k}^{t+1},O_{k}^{t+1})*\Theta_{k(\xi )}^{N(t+1)}(I_{k}^{t+1},L_{k}^{t+1},C_{k}^{t+1},A_{k}^{t+1},O_{k}^{t+1})}{\Theta_{k(\xi )}^{N(t)}(I_{k}^{t},L_{k}^{t},C_{k}^{t},A_{k}^{t},O_{k}^{t})*\Theta_{k(\xi )}^{N(t+1)}(I_{k}^{t},L_{k}^{t},C_{k}^{t},A_{k}^{t},O_{k}^{t})}}$$ (28)

$${\mathrm{CFNMPI}}_{k(\xi )}^{S1}=\sqrt{\frac{\Theta_{k(\xi )}^{S1(t)}(I_{k}^{t+1},L_{k}^{t+1},C_{k}^{t+1},A_{k}^{t+1},O_{k}^{t+1})*\Theta_{k(\xi )}^{S1(t+1)}(I_{k}^{t+1},L_{k}^{t+1},C_{k}^{t+1},A_{k}^{t+1},O_{k}^{t+1})}{\Theta_{k(\xi )}^{S1(t)}(I_{k}^{t},L_{k}^{t},C_{k}^{t},A_{k}^{t},O_{k}^{t})*\Theta_{k(\xi )}^{S1(t+1)}(I_{k}^{t},L_{k}^{t},C_{k}^{t},A_{k}^{t},O_{k}^{t})}}$$ (29)

$${\mathrm{CFNMPI}}_{k(\xi )}^{S2}=\sqrt{\frac{\Theta_{k(\xi )}^{S2(t)}(I_{k}^{t+1},L_{k}^{t+1},C_{k}^{t+1},A_{k}^{t+1},O_{k}^{t+1})*\Theta_{k(\xi )}^{S2(t+1)}(I_{k}^{t+1},L_{k}^{t+1},C_{k}^{t+1},A_{k}^{t+1},O_{k}^{t+1})}{\Theta_{k(\xi )}^{S2(t)}(I_{k}^{t},L_{k}^{t},C_{k}^{t},A_{k}^{t},O_{k}^{t})*\Theta_{k(\xi )}^{S2(t+1)}(I_{k}^{t},L_{k}^{t},C_{k}^{t},A_{k}^{t},O_{k}^{t})}}$$ (30)

**Step 6:** Analyze the CFNMPI values to understand the productivity change over time. It is crucial to note that the assessment of productivity change for the DMU under analysis depends on the value of the CFNMPI. This value can fall into three categories: greater than one, equal to one, or less than one:

- CFNMPI_*k*(*ξ*)_>1: If the CFNMPI exceeds one, it signifies a boost in productivity during the examined timeframe. This implies that the DMU has witnessed favorable enhancements in both efficiency and technology, ultimately leading to an upsurge in productivity when compared to the base period.
- CFNMPI_*k*(*ξ*)_ = 1: When the CFNMPI value is one, it indicates that there has been no alteration in productivity during the examined timeframe. This implies that the efficiency and technology of the DMU have consistently remained unchanged, leading to the maintenance of the same level of productivity as in the reference period.
- CFNMPI_*k*(*ξ*)_<1: If the CFNMPI falls below one, it indicates a drop in productivity during the evaluated period. This suggests that the DMU has encountered unfavorable developments in efficiency and technology, leading to reduced productivity when compared to the reference period.

## 6. A real-life application: Mutual funds

**Fig 2:**
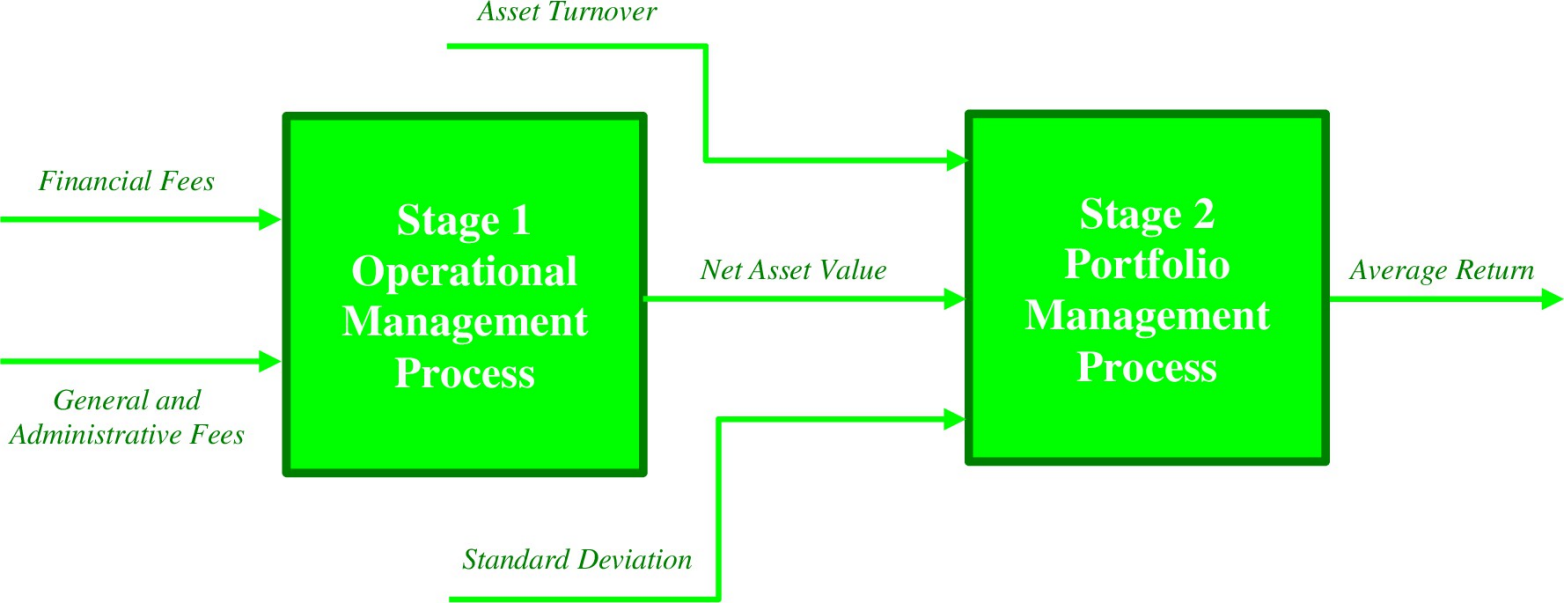
The graphical presentation of mutual funds structure.

In this section, the proposed credibility-based fuzzy network Malmquist productivity index will be implemented for 10 mutual funds from Iranian capital market. Mutual funds hold a crucial position in the capital market as significant financial institutions. They effectively manage the funds entrusted by investors, directing them towards specified investment plans. As a result, each individual who invests in these funds is entitled to a portion of the investment gains and is also exposed to potential risks. These distributions and vulnerabilities are commensurate with the respective interests that investors hold in the mutual funds. It should be noted that the functioning of mutual funds can be perceived as a dual-phase procedure. Initially, the management of mutual funds endeavors to allure investments from individuals, constituting the first stage (Operational Management). Subsequently, their attention shifts to the creation of the most advantageous portfolio, which forms the second stage (Portfolio Management). A visual representation of the empirical framework illustrating the activities of mutual funds is presented in Fig 2.

Tables 3 and 4 display the data sets related to the 10 Iranian mutual funds (IMFs) for two consecutive time periods. Accordingly, the results of the proposed credibility-based fuzzy NDEA approach for $\Theta_{k(\xi )}^{t}(I_{k}^{t},L_{k}^{t},C_{k}^{t},A_{k}^{t},O_{k}^{t})$, $\Theta_{k(\xi )}^{t+1}(I_{k}^{t+1},L_{k}^{t+1},C_{k}^{t+1},A_{k}^{t+1},O_{k}^{t+1})$, $\Theta_{k(\xi )}^{t}(I_{k}^{t+1},L_{k}^{t+1},C_{k}^{t+1},A_{k}^{t+1},O_{k}^{t+1})$, and $\Theta_{k(\xi )}^{t+1}(I_{k}^{t},L_{k}^{t},C_{k}^{t},A_{k}^{t},O_{k}^{t})$ are introduced in Tables 5 to 8, respectively. Finally, the results for efficiency change, technological change, and CFNMPI of IMFs at different confidence levels, including 0%, 20%, 40%, 60%, 80%, and 100%, are calculated in Tables 9 to 11, respectively:

**Table 3:** Data set of 10 Iranian mutual funds: Inputs and intermediate measure.

	IMFs	First Period	Second Period
**Financial Fees**	IMF-01	(0.07253, 0.08059, 0.08865)	(0.07180, 0.07978, 0.08776)
	IMF-02	(0.11000, 0.12222, 0.13444)	(0.06434, 0.07149, 0.07864)
	IMF-03	(0.08554, 0.09504, 0.10454)	(0.12562, 0.13958, 0.15354)
	IMF-04	(4.86189, 5.40210, 5.94231)	(4.67739, 5.19710, 5.71681)
	IMF-05	(0.01013, 0.01125, 0.01238)	(0.01441, 0.01601, 0.01761)
	IMF-06	(0.15918, 0.17687, 0.19456)	(0.11399, 0.12666, 0.13933)
	IMF-07	(0.04109, 0.04565, 0.05022)	(0.05657, 0.06285, 0.06914)
	IMF-08	(0.01396, 0.01551, 0.01706)	(0.12263, 0.13626, 0.14989)
	IMF-09	(0.14192, 0.15769, 0.17346)	(0.10268, 0.11409, 0.12550)
	IMF-10	(0.14643, 0.16270, 0.17897)	(0.05698, 0.06331, 0.06964)
**General and Administrative Fees**	IMF-01	(0.13312, 0.14791, 0.16270)	(0.18823, 0.20914, 0.23005)
	IMF-02	(0.09402, 0.10447, 0.11492)	(0.08899, 0.09888, 0.10877)
	IMF-03	(0.39022, 0.43358, 0.47694)	(0.51054, 0.56727, 0.62400)
	IMF-04	(4.42152, 4.91280, 5.40408)	(5.21820, 5.79800, 6.37780)
	IMF-05	(0.09010, 0.10011, 0.11012)	(0.05250, 0.05833, 0.06416)
	IMF-06	(0.35087, 0.38986, 0.42885)	(0.41967, 0.46630, 0.51293)
	IMF-07	(0.47599, 0.52888, 0.58177)	(0.56408, 0.62676, 0.68944)
	IMF-08	(0.29500, 0.32778, 0.36056)	(0.29482, 0.32758, 0.36034)
	IMF-09	(0.40016, 0.44462, 0.48908)	(0.44488, 0.49431, 0.54374)
	IMF-10	(0.35546, 0.39496, 0.43446)	(0.46208, 0.51342, 0.56476)
**Net Asset Value**	IMF-01	(5.72351, 6.35945, 6.99540)	(7.68142, 8.53491, 9.38840)
	IMF-02	(8.75240, 9.72489, 10.69738)	(9.06135, 10.06817, 11.07499)
	IMF-03	(79.87287, 88.74763, 97.62239)	(89.60087, 99.55652, 109.51217)
	IMF-04	(149.17708, 165.75231, 182.32754)	(207.46887, 230.52097, 253.57307)
	IMF-05	(1.38320, 1.53689, 1.69058)	(1.37660, 1.52955, 1.68251)
	IMF-06	(8.50590, 9.45100, 10.39610)	(8.42149, 9.35721, 10.29293)
	IMF-07	(20.91749, 23.24165, 25.56582)	(23.66875, 26.29861, 28.92847)
	IMF-08	(33.44024, 37.15582, 40.87140)	(35.11915, 39.02128, 42.92341)
	IMF-09	(75.61157, 84.01286, 92.41415)	(74.92314, 83.24793, 91.57272)
	IMF-10	(54.67507, 60.75008, 66.82509)	(71.19703, 79.10781, 87.01859)

**Table 4:** Data set of 10 Iranian mutual funds: Additional inputs and output.

	IMFs	First Period	Second Period
**Asset Turnover**	IMF-01	(0.11817, 0.13129, 0.14442)	(0.03694, 0.04104, 0.04514)
	IMF-02	(0.01824, 0.02026, 0.02229)	(0.01587, 0.01763, 0.01939)
	IMF-03	(0.22599, 0.25110, 0.27621)	(0.13761, 0.15290, 0.16819)
	IMF-04	(0.17208, 0.19120, 0.21032)	(0.15036, 0.16707, 0.18377)
	IMF-05	(0.25624, 0.28471, 0.31318)	(0.18438, 0.20487, 0.22536)
	IMF-06	(0.19006, 0.21118, 0.23230)	(0.07117, 0.07907, 0.08698)
	IMF-07	(0.10672, 0.11858, 0.13044)	(0.14234, 0.15816, 0.17397)
	IMF-08	(0.14067, 0.15631, 0.17194)	(0.07338, 0.08154, 0.08969)
	IMF-09	(0.19526, 0.21696, 0.23866)	(0.15896, 0.17662, 0.19428)
	IMF-10	(0.18893, 0.20992, 0.23091)	(0.17228, 0.19143, 0.21057)
**Standard Deviation**	IMF-01	(0.10708, 0.11898, 0.13088)	(0.06234, 0.06927, 0.07620)
	IMF-02	(0.20383, 0.22648, 0.24912)	(0.21509, 0.23899, 0.26289)
	IMF-03	(0.20527, 0.22807, 0.25088)	(0.21643, 0.24048, 0.26453)
	IMF-04	(0.09773, 0.10859, 0.11945)	(0.10392, 0.11547, 0.12701)
	IMF-05	(0.09751, 0.10834, 0.11917)	(0.10087, 0.11208, 0.12329)
	IMF-06	(0.29053, 0.32281, 0.35509)	(0.10920, 0.12133, 0.13347)
	IMF-07	(0.10480, 0.11644, 0.12809)	(0.10100, 0.11222, 0.12344)
	IMF-08	(0.13830, 0.15366, 0.16903)	(0.14995, 0.16661, 0.18327)
	IMF-09	(0.07395, 0.08216, 0.09038)	(0.06157, 0.06841, 0.07525)
	IMF-10	(0.14047, 0.15607, 0.17168)	(0.08690, 0.09656, 0.10622)
**Average Return**	IMF-01	(0.62878, 0.69864, 0.76851)	(0.45128, 0.50143, 0.55157)
	IMF-02	(0.84281, 0.93646, 1.03010)	(0.06019, 0.06688, 0.07357)
	IMF-03	(0.59445, 0.66050, 0.72655)	(0.26724, 0.29693, 0.32662)
	IMF-04	(0.09738, 0.10819, 0.11901)	(0.11074, 0.12304, 0.13534)
	IMF-05	(0.21775, 0.24195, 0.26614)	(0.35957, 0.39952, 0.43948)
	IMF-06	(0.23781, 0.26424, 0.29066)	(0.97775, 1.08638, 1.19502)
	IMF-07	(0.19986, 0.22207, 0.24428)	(0.33304, 0.37004, 0.40704)
	IMF-08	(0.10293, 0.11436, 0.12580)	(0.05274, 0.05860, 0.06446)
	IMF-09	(0.14453, 0.16059, 0.17665)	(0.38791, 0.43101, 0.47411)
	IMF-10	(0.24263, 0.26959, 0.29655)	(0.46413, 0.51570, 0.56727)

**Table 5:** The results of CFNDEA approach: $\Theta_{k(\xi )}^{t}(I_{k}^{t},L_{k}^{t},C_{k}^{t},A_{k}^{t},O_{k}^{t})$.

	IMFs	CL = 0%	CL = 20%	CL = 40%	CL = 60%	CL = 80%	CL = 100%
**Overall**	IMF-01	1.4935630	1.2713620	1.0830540	0.9228717	0.7861756	0.6692059
	IMF-02	1.4936980	1.2714910	1.0831790	0.9229913	0.7862904	0.6693158
	IMF-03	0.7372800	0.6432560	0.5681137	0.5012538	0.4416361	0.3883859
	IMF-04	0.2530366	0.2153765	0.1834630	0.1563178	0.1331543	0.1133349
	IMF-05	0.5681189	0.4836053	0.4119826	0.3510570	0.2990641	0.2545738
	IMF-06	0.2396321	0.2039825	0.1737707	0.1480711	0.1261396	0.1073729
	IMF-07	0.4974556	0.4234543	0.3607408	0.3073939	0.2618688	0.2229132
	IMF-08	0.6921076	0.6138184	0.5439371	0.4814236	0.4254022	0.3751304
	IMF-09	0.6608846	0.5856354	0.5184960	0.4584691	0.4047136	0.3565147
	IMF-10	0.6128615	0.5397875	0.4750909	0.4176831	0.3666516	0.3212257
**First Stage**	IMF-01	0.3131779	0.2666319	0.2271769	0.1936078	0.1649553	0.1404330
	IMF-02	0.6755345	0.5752977	0.4902972	0.4179492	0.3561758	0.3032899
	IMF-03	1.4938271	1.2716161	1.0832987	0.9231066	0.7864009	0.6694215
	IMF-04	0.2449436	0.2085919	0.1777671	0.1515319	0.1291319	0.1099555
	IMF-05	0.1550329	0.1319713	0.1124273	0.0958022	0.0816145	0.0694741
	IMF-06	0.1766747	0.1504101	0.1281481	0.1092083	0.0930432	0.0792089
	IMF-07	0.4896169	0.4167849	0.3550619	0.3025574	0.2577507	0.2194096
	IMF-08	1.4938272	1.2716162	1.0832986	0.9231064	0.7864008	0.6694214
	IMF-09	1.3790153	1.1738830	1.0000390	0.8521589	0.7259601	0.6179716
	IMF-10	1.1225507	0.9555682	0.8140551	0.6936773	0.5909486	0.5030431
**Second Stage**	IMF-01	1.4938267	1.2716165	1.0832984	0.9231065	0.7864009	0.6694215
	IMF-02	1.4938276	1.2716159	1.0832981	0.9231065	0.7864008	0.6694215
	IMF-03	0.7367853	0.0903839	0.0769988	0.0656127	0.0558960	0.0475814
	IMF-04	0.2530974	0.2154341	0.1835177	0.1563696	0.1332035	0.1133813
	IMF-05	0.5681640	0.4836487	0.4120244	0.3510971	0.2991025	0.2546106
	IMF-06	0.2396692	0.2040183	0.1738051	0.1481041	0.1261712	0.1074031
	IMF-07	0.4974597	0.4234582	0.3607445	0.3073976	0.2618724	0.2229165
	IMF-08	0.0437435	0.0372366	0.0317221	0.0270312	0.0230281	0.0196026
	IMF-09	0.0305149	0.0259756	0.0221287	0.0188563	0.0160637	0.0136741
	IMF-10	0.0632011	0.0537998	0.0458325	0.0390551	0.0332713	0.0283222

**Table 6:** The results of CFNDEA approach: $\Theta_{k(\xi )}^{t+1}(I_{k}^{t+1},L_{k}^{t+1},C_{k}^{t+1},A_{k}^{t+1},O_{k}^{t+1})$.

	IMFs	CL = 0%	CL = 20%	CL = 40%	CL = 60%	CL = 80%	CL = 100%
**Overall**	IMF-01	1.3281040	1.1305130	0.9630625	0.8206223	0.6990674	0.5950542
	IMF-02	0.5415394	0.4738321	0.4143803	0.3620516	0.3159024	0.2751409
	IMF-03	0.6889390	0.6111535	0.5416987	0.4795468	0.4238324	0.3738215
	IMF-04	0.2662410	0.2304113	0.1993413	0.1723360	0.1488187	0.1283072
	IMF-05	0.6802621	0.5592452	0.4600390	0.3784585	0.3130688	0.2664971
	IMF-06	1.4930300	1.2708480	1.0825590	0.9223961	0.7857195	0.6687691
	IMF-07	0.5500126	0.4681901	0.3988485	0.3398637	0.2895274	0.2464550
	IMF-08	0.5580786	0.4920686	0.4334614	0.3813335	0.3349036	0.2935063
	IMF-09	1.0506410	0.8943558	0.7619112	0.6492494	0.5531071	0.4708392
	IMF-10	0.8907938	0.7583034	0.6460227	0.5505121	0.4690052	0.3992597
**First Stage**	IMF-01	0.3470348	0.2954339	0.2516990	0.2144924	0.1827380	0.1555634
	IMF-02	0.8666868	0.7377647	0.6285069	0.5355669	0.4562531	0.3883841
	IMF-03	1.4938270	1.2716161	1.0832985	0.9231065	0.7864009	0.6694215
	IMF-04	0.3384027	0.2880640	0.2454035	0.2091144	0.1781457	0.1516458
	IMF-05	0.2231990	0.1899975	0.1618602	0.1379252	0.1174825	0.1000078
	IMF-06	0.1707749	0.1453737	0.1238464	0.1055339	0.0899061	0.0765330
	IMF-07	0.4993330	0.4251157	0.3622057	0.3086816	0.2629969	0.2238982
	IMF-08	1.0139212	0.8630975	0.7352788	0.6265498	0.5337622	0.4543635
	IMF-09	1.4555608	1.2390421	1.0555486	0.8994600	0.7662560	0.6522735
	IMF-10	1.4938273	1.2716161	1.0832987	0.9231065	0.7864008	0.6694212
**Second Stage**	IMF-01	1.3283693	1.1307693	0.9633086	0.8208587	0.6992942	0.5952714
	IMF-02	0.0915790	0.0779564	0.0664115	0.0565910	0.0482102	0.0410388
	IMF-03	0.0412135	0.0350829	0.0298874	0.0254679	0.0216963	0.0184689
	IMF-04	0.0092578	0.0078805	0.0067133	0.0057205	0.0048732	0.0041482
	IMF-05	1.4938270	1.2716161	1.0832988	0.9231065	0.3130927	0.2665200
	IMF-06	1.4938273	1.2716166	1.0832989	0.9231065	0.7864008	0.6694215
	IMF-07	0.5500419	0.4682183	0.3988756	0.3398897	0.2895524	0.2464789
	IMF-08	0.0085899	0.0073123	0.0062295	0.0053084	0.0045223	0.0038497
	IMF-09	1.0503859	0.8941102	0.7616747	0.6490222	0.5528893	0.4706306
	IMF-10	0.8904423	0.7579647	0.6456967	0.5501989	0.4687048	0.3989720

**Table 7:** The results of CFNDEA approach: $\Theta_{k(\xi )}^{t}(I_{k}^{t+1},L_{k}^{t+1},C_{k}^{t+1},A_{k}^{t+1},O_{k}^{t+1})$.

	IMFs	CL = 0%	CL = 20%	CL = 40%	CL = 60%	CL = 80%	CL = 100%
**Overall**	IMF-01	2.1635060	1.8416080	1.5688130	1.3367630	1.1387400	0.9692934
	IMF-02	0.4991747	0.4351636	0.3792129	0.3301854	0.2871365	0.2492762
	IMF-03	0.6459524	0.5710794	0.5044710	0.4450894	0.3920609	0.3446450
	IMF-04	0.2706901	0.2304218	0.1962978	0.1672719	0.1425030	0.1213093
	IMF-05	0.9068141	0.7719172	0.6575961	0.5603491	0.4773601	0.4063465
	IMF-06	2.6218770	2.2316670	1.9009850	1.6197000	1.3796650	1.1742770
	IMF-07	0.8384669	0.7137077	0.6079801	0.5180451	0.4412980	0.3756271
	IMF-08	0.5157394	0.4530506	0.3976570	0.3486160	0.3051337	0.2665365
	IMF-09	1.6014060	1.3631180	1.1611830	0.9894139	0.8428351	0.7174128
	IMF-10	1.3578660	1.1558550	0.9846600	0.8390373	0.7147682	0.6084337
**First Stage**	IMF-01	0.2975459	0.2533041	0.2158064	0.1839058	0.1566798	0.1333805
	IMF-02	0.7431125	0.6325724	0.5388928	0.4592044	0.3911995	0.3330074
	IMF-03	1.2808333	1.0903057	0.9288389	0.7914875	0.6742736	0.5739735
	IMF-04	0.2889965	0.2460841	0.2097008	0.1787386	0.1523058	0.1296792
	IMF-05	0.1913116	0.1628576	0.1387427	0.1182288	0.1007220	0.0857408
	IMF-06	0.1464056	0.1246304	0.1061759	0.0904770	0.0770795	0.0656149
	IMF-07	0.4429827	0.3770878	0.3212437	0.2737400	0.2332011	0.1985117
	IMF-08	0.8693538	0.7400349	0.6304408	0.5372147	0.4576570	0.3895792
	IMF-09	1.2290137	1.0462002	0.8912696	0.7594772	0.6470066	0.5507645
	IMF-10	1.4971813	1.2744714	1.0857311	0.9251792	0.7881666	0.6709245
**Second Stage**	IMF-01	2.1640947	1.8421760	1.5693590	1.3372883	1.1392423	0.9697750
	IMF-02	0.1033089	0.0879414	0.0749179	0.0638395	0.0543853	0.0462953
	IMF-03	0.0427029	0.0363508	0.0309675	0.0263883	0.0224804	0.0191364
	IMF-04	0.2705281	0.2302647	0.1961457	0.1671250	0.1423615	0.1211732
	IMF-05	0.9068770	0.7719778	0.6576544	0.5604052	0.4774140	0.4063980
	IMF-06	2.6236186	2.2333444	1.9025992	1.6212512	1.3811531	1.1757010
	IMF-07	0.8387253	0.7139567	0.6082196	0.5182753	0.4415187	0.3758385
	IMF-08	0.0214684	0.0182749	0.0155685	0.0132664	0.0113017	0.0096206
	IMF-09	1.6016834	1.3633850	1.1614405	0.9896612	0.8430723	0.7176399
	IMF-10	1.3577852	1.1557765	0.9845848	0.8389651	0.7146989	0.6083673

**Table 8:** The results of CFNDEA approach: $\Theta_{k(\xi )}^{t+1}(I_{k}^{t},L_{k}^{t},C_{k}^{t},A_{k}^{t},O_{k}^{t})$.

	IMFs	CL = 0%	CL = 20%	CL = 40%	CL = 60%	CL = 80%	CL = 100%
**Overall**	IMF-01	0.9795461	0.8338236	0.7103284	0.6052779	0.5156298	0.4389182
	IMF-02	5.0245940	4.2770940	3.6436140	3.1047500	2.6448950	2.2514010
	IMF-03	0.7742852	0.6870657	0.6093335	0.5398671	0.4776476	0.4218180
	IMF-04	0.2338894	0.2019716	0.1743757	0.1504577	0.1296848	0.1116135
	IMF-05	0.3808806	0.3171594	0.2701887	0.2302331	0.1961357	0.1669584
	IMF-06	0.2253641	0.1907910	0.1616933	0.1371049	0.1162542	0.0985205
	IMF-07	0.4427777	0.3842707	0.3334292	0.2891293	0.2504430	0.2165981
	IMF-08	0.8689775	0.7812681	0.7018522	0.6297678	0.5642027	0.5044671
	IMF-09	0.7025193	0.6245586	0.5547677	0.4921549	0.4359472	0.3854808
	IMF-10	0.6516928	0.5761323	0.5089562	0.4490978	0.3956619	0.3478919
**First Stage**	IMF-01	0.3652074	0.3109318	0.2649241	0.2257793	0.1923671	0.1637711
	IMF-02	0.7872783	0.6704999	0.5714634	0.4871626	0.4151782	0.3535464
	IMF-03	1.7900459	1.5237717	1.2981115	1.1061543	0.9423405	0.8021646
	IMF-04	0.2871644	0.2444476	0.2082463	0.1774518	0.1511722	0.1286847
	IMF-05	0.1633216	0.1389281	0.1183603	0.1008629	0.0859299	0.0731507
	IMF-06	0.2063427	0.1756487	0.1496364	0.1275089	0.1086257	0.0924674
	IMF-07	0.6086653	0.5181246	0.4413940	0.3761230	0.3204218	0.2727581
	IMF-08	2.8639518	2.4379314	2.0768906	1.7697717	1.5076811	1.2834092
	IMF-09	1.6083358	1.3690914	1.1663385	0.9938668	0.8466823	0.7207357
	IMF-10	1.3092231	1.1144724	0.9494268	0.8090310	0.6892190	0.5866959
**Second Stage**	IMF-01	0.9796639	0.8339370	0.7104375	0.6053826	0.5157303	0.4390144
	IMF-02	5.0251698	4.2776488	3.6441476	3.1052614	2.6453866	2.2518704
	IMF-03	0.1014386	0.0863494	0.0735617	0.0626839	0.0534009	0.0454574
	IMF-04	0.0112732	0.0095961	0.0081748	0.0069658	0.0059341	0.0050513
	IMF-05	0.9240318	0.3171803	0.2702088	0.2302525	0.1961542	0.1669762
	IMF-06	0.3160069	0.2689997	0.2291626	0.1952751	0.1663559	0.1416099
	IMF-07	0.1271797	0.1082614	0.0922287	0.0785904	0.0669518	0.0569925
	IMF-08	0.0176041	0.0149856	0.0127664	0.0108786	0.0092677	0.0078891
	IMF-09	0.0327235	0.0278557	0.0237304	0.0202212	0.0057571	0.0049009
	IMF-10	0.0600875	0.0511494	0.0435745	0.0371310	0.0316322	0.0269268

**Table 9:** The results of efficiency change of IMFs based on the CFNDEA approach.

	IMFs	CL = 0%	CL = 20%	CL = 40%	CL = 60%	CL = 80%	CL = 100%
**Overall**	IMF-01	0.8892186	0.8892141	0.8892100	0.8892052	0.8892001	0.8891945
	IMF-02	0.3625495	0.3726586	0.3825594	0.3922590	0.4017630	0.4110778
	IMF-03	0.9344333	0.9500937	0.9535040	0.9566946	0.9596869	0.9625002
	IMF-04	1.0521838	1.0698071	1.0865477	1.1024720	1.1176410	1.1321067
	IMF-05	1.1973939	1.1564083	1.1166467	1.0780543	1.0468284	1.0468363
	IMF-06	6.2305092	6.2301815	6.2298132	6.2294134	6.2289677	6.2284720
	IMF-07	1.1056516	1.1056449	1.1056373	1.1056293	1.1056201	1.1056097
	IMF-08	0.8063466	0.8016518	0.7968962	0.7920956	0.7872634	0.7824114
	IMF-09	1.5897496	1.5271546	1.4694640	1.4161247	1.3666630	1.3206726
	IMF-10	1.4534994	1.4048184	1.3597876	1.3180138	1.2791577	1.2429258
**First Stage**	IMF-01	1.1081075	1.1080217	1.1079429	1.1078707	1.1078033	1.1077414
	IMF-02	1.2829644	1.2824051	1.2818897	1.2814160	1.2809773	1.2805704
	IMF-03	1.0000000	1.0000000	0.9999999	0.9999999	1.0000000	1.0000000
	IMF-04	1.3815535	1.3809933	1.3804775	1.3800027	1.3795637	1.3791560
	IMF-05	1.4396881	1.4396874	1.4396873	1.4396874	1.4394803	1.4394974
	IMF-06	0.9666068	0.9665155	0.9664316	0.9663544	0.9662831	0.9662171
	IMF-07	1.0198442	1.0199882	1.0201199	1.0202413	1.0203537	1.0204575
	IMF-08	0.6787407	0.6787406	0.6787407	0.6787406	0.6787406	0.6787407
	IMF-09	1.0555074	1.0555073	1.0555074	1.0555073	1.0555071	1.0555074
	IMF-10	1.3307436	1.3307434	1.3307437	1.3307434	1.3307433	1.3307433
**Second Stage**	IMF-01	0.8892392	0.8892377	0.8892366	0.8892351	0.8892338	0.8892325
	IMF-02	0.0613049	0.0613050	0.0613049	0.0613049	0.0613049	0.0613049
	IMF-03	0.0559370	0.3881547	0.3881545	0.3881545	0.3881543	0.3881541
	IMF-04	0.0365781	0.0365798	0.0365814	0.0365830	0.0365845	0.0365859
	IMF-05	2.6292182	2.6292143	2.6292106	2.6292056	1.0467738	1.0467750
	IMF-06	6.2328706	6.2328557	6.2328390	6.2328220	6.2328093	6.2327931
	IMF-07	1.1057014	1.1057013	1.1057010	1.1057005	1.1057003	1.1057003
	IMF-08	0.1963700	0.1963728	0.1963758	0.1963790	0.1963825	0.1963863
	IMF-09	34.4220885	34.4211838	34.4202672	34.4193884	34.4185156	34.4176484
	IMF-10	14.0890353	14.0886173	14.0881909	14.0877703	14.0873431	14.0869062

**Table 10:** The results of technological change of IMFs based on the CFNDEA approach.

	IMFs	CL = 0%	CL = 20%	CL = 40%	CL = 60%	CL = 80%	CL = 100%
**Overall**	IMF-01	1.5760222	1.5760077	1.5759913	1.5759731	1.5759540	1.5759319
	IMF-02	0.5234704	0.5225120	0.5215860	0.5206899	0.5198224	0.5189816
	IMF-03	0.9448777	0.9353322	0.9318142	0.9283111	0.9248220	0.9213466
	IMF-04	1.0487817	1.0326756	1.0178654	1.0042007	0.9915539	0.9798171
	IMF-05	1.4100879	1.4507446	1.4763459	1.5025369	1.5247793	1.5247714
	IMF-06	1.3664760	1.3702045	1.3737440	1.3771055	1.3803031	1.3833471
	IMF-07	1.3087025	1.2960857	1.2842114	1.2730134	1.2624337	1.2524206
	IMF-08	0.8579264	0.8505120	0.8432000	0.8359776	0.8288338	0.8217588
	IMF-09	1.1974496	1.1954698	1.1934804	1.1914816	1.1893882	1.1870953
	IMF-10	1.1972906	1.1950346	1.1927994	1.1905844	1.1883885	1.1862108
**First Stage**	IMF-01	0.8574647	0.8574611	0.8574578	0.8574546	0.8574519	0.8574491
	IMF-02	0.8577404	0.8577153	0.8576926	0.8576715	0.8576521	0.8576340
	IMF-03	0.8458906	0.8458906	0.8458906	0.8458906	0.8458905	0.8458906
	IMF-04	0.8534874	0.8537940	0.8540763	0.8543367	0.8545773	0.8548010
	IMF-05	0.9020167	0.9023495	0.9023353	0.9023224	0.9023753	0.9023586
	IMF-06	0.8567605	0.8568112	0.8568579	0.8569011	0.8569407	0.8569777
	IMF-07	0.8447675	0.8447079	0.8446533	0.8446032	0.8445568	0.8445138
	IMF-08	0.6687496	0.6687494	0.6687494	0.6687494	0.6687494	0.6687494
	IMF-09	0.8508625	0.8508648	0.8508668	0.8508689	0.8508706	0.8508721
	IMF-10	0.9270073	0.9270075	0.9270074	0.9270074	0.9270076	0.9270073
**Second Stage**	IMF-01	1.5761236	1.5761226	1.5761210	1.5761199	1.5761181	1.5761170
	IMF-02	0.5790896	0.5790904	0.5790914	0.5790926	0.5790936	0.5790944
	IMF-03	2.7433277	1.0414174	1.0414180	1.0414186	1.0414194	1.0414202
	IMF-04	25.6137329	25.6121320	25.6105880	25.6090906	25.6076299	25.6062119
	IMF-05	0.6109665	0.9621357	0.9621360	0.9621364	1.5248332	1.5248315
	IMF-06	1.1541387	1.1541398	1.1541410	1.1541424	1.1541435	1.1541447
	IMF-07	2.4422049	2.4421945	2.4421842	2.4421743	2.4421640	2.4421537
	IMF-08	2.4920389	2.4920137	2.4919867	2.4919587	2.4919274	2.4918933
	IMF-09	1.1924500	1.1924479	1.1924462	1.1924439	2.0626939	2.0626554
	IMF-10	1.2664347	1.2664341	1.2664340	1.2664335	1.2664334	1.2664336

**Table 11:** The results of credibility-based fuzzy network Malmquist productivity index.

	IMFs	CL = 0%	CL = 20%	CL = 40%	CL = 60%	CL = 80%	CL = 100%
**Overall**	IMF-01	1.4014283	1.4014083	1.4013873	1.4013635	1.4013384	1.4013100
	IMF-02	0.1897839	0.1947186	0.1995376	0.2042453	0.2088454	0.2133418
	IMF-03	0.8829253	0.8886533	0.8884886	0.8881102	0.8875396	0.8867962
	IMF-04	1.1035111	1.1047636	1.1059593	1.1071031	1.1082012	1.1092575
	IMF-05	1.6884306	1.6776531	1.6485568	1.6198163	1.5961823	1.5961861
	IMF-06	8.5138415	8.5366230	8.5581687	8.5785598	8.5978634	8.6161388
	IMF-07	1.4469691	1.4330106	1.4198721	1.4074809	1.3957720	1.3846884
	IMF-08	0.6917860	0.6818144	0.6719429	0.6621742	0.6525106	0.6429534
	IMF-09	1.9036450	1.8256673	1.7537764	1.6872865	1.6254929	1.5677643
	IMF-10	1.7402611	1.6788066	1.6219538	1.5692067	1.5201363	1.4743720
**First Stage**	IMF-01	0.9501631	0.9500855	0.9500143	0.9499488	0.9498881	0.9498319
	IMF-02	1.1004504	1.0999384	1.0994673	1.0990340	1.0986329	1.0982607
	IMF-03	0.8458906	0.8458906	0.8458905	0.8458905	0.8458905	0.8458905
	IMF-04	1.1791386	1.1790837	1.1790332	1.1789870	1.1789438	1.1789039
	IMF-05	1.2986227	1.2991013	1.2990807	1.2990621	1.2989514	1.2989428
	IMF-06	0.8281505	0.8281213	0.8280945	0.8280701	0.8280473	0.8280265
	IMF-07	0.8615313	0.8615921	0.8616476	0.8616991	0.8617467	0.8617905
	IMF-08	0.4539075	0.4539074	0.4539074	0.4539074	0.4539073	0.4539074
	IMF-09	0.8980916	0.8980940	0.8980962	0.8980984	0.8981000	0.8981018
	IMF-10	1.2336091	1.2336091	1.2336092	1.2336090	1.2336091	1.2336088
**Second Stage**	IMF-01	1.4015510	1.4015476	1.4015445	1.4015411	1.4015375	1.4015344
	IMF-02	0.0355011	0.0355011	0.0355012	0.0355012	0.0355013	0.0355013
	IMF-03	0.1534534	0.4042311	0.4042311	0.4042313	0.4042314	0.4042315
	IMF-04	0.9369007	0.9368868	0.9368718	0.9368571	0.9368422	0.9368272
	IMF-05	1.6063643	2.5296609	2.5296581	2.5296543	1.5961556	1.5961554
	IMF-06	7.1935970	7.1935865	7.1935751	7.1935644	7.1935565	7.1935454
	IMF-07	2.7003495	2.7003377	2.7003255	2.7003133	2.7003014	2.7002901
	IMF-08	0.4893618	0.4893637	0.4893659	0.4893684	0.4893708	0.4893738
	IMF-09	41.0466186	41.0454674	41.0443154	41.0431914	70.9948631	70.9917490
	IMF-10	17.8428429	17.8423051	17.8417633	17.8412242	17.8406821	17.8401314

According to the results in in Table 11, it is clear that only two mutual funds, IMF-05 and IMF-10, have significantly improved their productivity in all stages, including overall and stages 1 and 2, during the specified period. As a result, by analyzing the strategies, practices, and processes implemented by IMF-05 and IMF-10, other mutual funds can identify areas where they can make improvements. This could include factors such as investment selection, risk management, portfolio diversification, and operational efficiency. Benchmarking allows mutual funds to identify best practices and set performance goals based on the success of top-performing funds. By learning from the achievements of IMF-05 and IMF-10, other funds can aim to enhance their productivity and ultimately deliver better results for their investors. The findings of this research will contribute to the existing literature on productivity assessment in two-stage network systems. Moreover, they will provide valuable insights for decision-makers in the mutual fund industry, enabling them to identify areas of improvement and implement strategies to enhance efficiency and productivity. Notably, the practical application of the uncertain network Malmquist productivity index results for policymakers in the context of mutual funds could involve:

- **Performance Assessment:** Policymakers can utilize the Malmquist Productivity Index to evaluate the efficiency and productivity changes over time in mutual funds. This can help identify underperforming funds that may require intervention or support.
- **Resource Allocation:** By analyzing the productivity changes using the index, policymakers can make informed decisions on resource allocation within the mutual fund industry. Funds showing productivity improvements could be encouraged or incentivized, while those lagging behind may require attention.
- **Regulatory Oversight:** The Malmquist Productivity Index results can assist policymakers in setting regulatory standards and benchmarks for mutual funds. By comparing fund performance using this index, policymakers can implement regulations that promote efficiency and competitiveness in the industry.
- **Policy Formulation:** Policymakers can use the insights from the Malmquist Productivity Index to develop policies aimed at enhancing overall productivity and innovation within the mutual fund sector. This could involve introducing measures to support technological advancements, streamline operations, or foster collaboration among funds for mutual benefit.
- **Investor Protection:** Policymakers can leverage the index results to ensure investor protection by monitoring the productivity and efficiency of mutual funds. This can help in safeguarding investor interests and maintaining the stability and transparency of the market.
- By incorporating the findings from the uncertain network Malmquist productivity index analysis into policy decisions, policymakers can work towards fostering a more efficient, competitive, and sustainable mutual fund industry.

## 7. Conclusions and future research directions

This research paper investigated the application of the Malmquist productivity index in analyzing the productivity change of two-stage network systems, specifically focusing on mutual funds. The study utilized the fuzzy network data envelopment analysis approach and incorporated the principles of credibility theory to account for data uncertainty. The findings of this research highlight the significance of considering the specific characteristics of mutual funds as two-stage decision-making units when assessing productivity change. The Malmquist productivity index proved to be a valuable tool in capturing and quantifying these changes over time. Moreover, by incorporating fuzzy set theory and credibility theory, the analysis accounted for data uncertainty and enhanced the accuracy of the results. The research contributes to the existing literature on productivity measurement by offering a novel approach for evaluating the performance of mutual funds within the context of two-stage network systems. The findings provide valuable insights for fund managers, investors, and policymakers in understanding the dynamics of mutual fund productivity and making informed decisions. Overall, this research contributes to the advancement of productivity measurement methods and offers a comprehensive and effective framework for analyzing the performance of two-stage network decision-making units under data ambiguity. Future research can build upon this study by expanding the application of the Malmquist productivity index to other sectors or industries with similar characteristics of two-stage network systems [69–71]. Furthermore, the Malmquist productivity index can be extended to address special data scenarios, including those involving negative data [72–74]. It is also suggested to use other commonly used approaches in the field of uncertain programming to deal with other types of uncertainty [75–77].

## Supporting information

S1 Appendix (DOCX)

## References

[1] Färe R., Grosskopf S., Norris M., & Zhang Z. (1994). Productivity growth, technical progress, and efficiency change in industrialized countries. *The American Economic Review*, 66–83.

[2] Coelli T. J., Rao D. S. P., O’Donnell C. J., & Battese G. E. (2005). *An Introduction to Efficiency and Productivity Analysis*. Springer Science & Business Media.

[3] Liu J., Jungyin K., Jaewoo S., Heechul L., & Shah W. U. H. (2024). Evaluating the efficiency, productivity change, and technology gaps of China’s provincial higher education systems: A comprehensive analytical framework. *Plos One*, 19(1), e0294902. doi: 10.1371/journal.pone.0294902 38241214 PMC10798458

[4] Liu J. S., Lu L. Y., Lu W. M., & Lin B. J. (2013). A survey of DEA applications. *Omega*, 41(5), 893–902.

[5] Emrouznejad A., & Yang G. L. (2018). A survey and Analysis of the First 40 Years of Scholarly Literature in DEA: 1978–2016. *Socio-Economic Planning Sciences*, 61, 4–8.

[6] Peykani P., Farzipoor Saen R., Seyed Esmaeili F. S., & Gheidar‐Kheljani J. (2021). Window data envelopment analysis approach: A review and bibliometric analysis. *Expert Systems*, 38(7), e12721.

[7] Charnes A., Cooper W. W., & Rhodes E. (1978). Measuring the efficiency of decision making units. *European Journal of Operational Research*, 2(6), 429–444.

[8] Banker R. D., Charnes A., & Cooper W. W. (1984). Some models for estimating technical and scale inefficiencies in data envelopment analysis. *Management Science*, 30(9), 1078–1092.

[9] Cooper W. W., Seiford L. M., & Zhu J. (2011). *Handbook on Data Envelopment Analysis*. Springer, New York, NY.

[10] Zhu W., & Zhou Z. (2013). Interval efficiency of two-stage network DEA model with imprecise data. *INFOR*: *Information Systems and Operational Research*, 51(3), 142–150.

[11] Esfandiari M., Hafezalkotob A., Khalili-Damghani K., & Amirkhan M. (2017). Robust two-stage DEA models under discrete uncertain data. *International Journal of Management Science and Engineering Management*, 12(3), 216–224.

[12] Izadikhah M., Azadi M., Toloo M., & Hussain F. K. (2021). Sustainably resilient supply chains evaluation in public transport: A fuzzy chance-constrained two-stage DEA approach. *Applied Soft Computing*, 113, 107879.

[13] Jiang B., Chen H., Li J., & Lio W. (2021). The uncertain two-stage network DEA models. *Soft Computing*, 25, 421–429.

[14] Izadikhah M., & Farzipoor Saen R. (2023). Developing a linear stochastic two-stage data envelopment analysis model for evaluating sustainability of supply chains: a case study in welding industry. *Annals of Operations Research*, 322(1), 195–215.

[15] Kao C., & Hwang S. N. (2014). Multi-Period Efficiency and Malmquist Productivity Index in Two-Stage Production Systems. *European Journal of Operational Research*, 232(3), 512–521.

[16] Lin T. X., Wu Z. H., Ji X. X., & Yang J. J. (2021). Research on the Operating Efficiency of Chinese Listed Pharmaceutical Companies Based on Two-Stage Network DEA and Malmquist. *Mathematical Problems in Engineering*, 2021, 1475781.

[17] Yu M. M., & Nguyen M. A. T. (2023). Productivity changes of Asia-Pacific airlines: A Malmquist productivity index approach for a two-stage dynamic system. *Omega*, 115, 102774.

[18] Castelli L., Pesenti R., & Ukovich W. (2010). A Classification of DEA Models When the Internal Structure of the Decision Making Units is Considered. *Annals of Operations Research*, 173(1), 207–235.

[19] Cook W. D., Liang L., & Zhu J. (2010). Measuring performance of two-stage network structures by DEA: a review and future perspective. *Omega*, 38(6), 423–430.

[20] Cook W. D., & Zhu J. (2014). *Data Envelopment Analysis*: *A Handbook of Modeling Internal Structure and Network*. Springer.

[21] Färe R., & Grosskopf S. (1992). Malmquist productivity indexes and Fisher ideal indexes. *The Economic Journal*, 102(410), 158–160.

[22] Kao C. (2010). Malmquist productivity index based on common-weights DEA: The case of Taiwan forests after reorganization. *Omega*, 38(6), 484–491.

[23] Zhu C., Zhu N., Emrouznejad A., & Ye T. (2023). A new Malmquist productivity index with an application to commercial banks. *IMA Journal of Management Mathematics*, 015.

[24] Emrouznejad A., Rostamy-Malkhalifeh M., Hatami-Marbini A., Tavana M., & Aghayi N. (2011). An overall profit Malmquist productivity index with fuzzy and interval data. *Mathematical and Computer Modelling*, 54(11–12), 2827–2838.

[25] Peykani P., Mohammadi E., Farzipoor Saen R., Sadjadi S. J., & Rostamy‐Malkhalifeh M. (2020). Data envelopment analysis and robust optimization: A review. *Expert Systems*, 37(4), e12534.

[26] Seyed Esmaeili F. S., Rostamy-Malkhalifeh M., & Hosseinzadeh Lotfi F. (2022). Interval Network Malmquist Productivity Index for Examining Productivity Changes of Insurance Companies under Data Uncertainty: A Case Study. *Journal of Mathematical Extension*, 16(8), 9.

[27] Emrouznejad A., & Tavana M. (2014). *Performance Measurement with Fuzzy Data Envelopment Analysis*, Springer.

[28] Lozano S. (2014). Process efficiency of two-stage systems with fuzzy data. *Fuzzy Sets and Systems*, 243, 36–49.

[29] Peykani P., Hosseinzadeh Lotfi F., Sadjadi S. J., Ebrahimnejad A., & Mohammadi E. (2022). Fuzzy chance-constrained data envelopment analysis: a structured literature review, current trends, and future directions. *Fuzzy Optimization and Decision Making*, 21(2), 197–261.

[30] Peykani P., Mohammadi E., Pishvaee M. S., Rostamy-Malkhalifeh M., & Jabbarzadeh A. (2018). A novel fuzzy data envelopment analysis based on robust possibilistic programming: Possibility, necessity and credibility-based approaches. *RAIRO-Operations Research*, 52(4–5), 1445–1463.

[31] Mahla D., & Agarwal S. (2021). A credibility approach on fuzzy slacks based measure (SBM) DEA model. *Iranian Journal of Fuzzy Systems*, 18(3), 39–49.

[32] Peykani P., Memar-Masjed E., Arabjazi N., & Mirmozaffari M. (2022). Dynamic performance assessment of hospitals by applying credibility-based fuzzy window data envelopment analysis. *Healthcare*, 10(5), 876. doi: 10.3390/healthcare10050876 35628013 PMC9141957

[33] Vidal-García J., Vidal M., Boubaker S., & Hassan M. (2018). The efficiency of mutual funds. *Annals of Operations Research*, 267, 555–584.

[34] Lin R., & Liu Q. (2021). Multiplier dynamic data envelopment analysis based on directional distance function: An application to mutual funds. *European Journal of Operational Research*, 293(3), 1043–1057.

[35] Daniel D., & John R. (2024). Assessing Mutual Fund Success: A Comprehensive Literature Review of Efficiency Measurement through Data Envelopment Analysis (DEA). *Ajasra*, 13(2), 239–255.

[36] Basso A., & Funari S. (2001). A data envelopment analysis approach to measure the mutual fund performance. *European Journal of Operational Research*, 135(3), 477–492.

[37] Basso A., & Funari S. (2016). DEA performance assessment of mutual funds. *Data Envelopment Analysis*: *A Handbook of Empirical Studies and Applications*, 229–287. Springer, Boston, MA.

[38] Kaffash S., & Marra M. (2017). Data envelopment analysis in financial services: a citations network analysis of banks, insurance companies and money market funds. *Annals of Operations Research*, 253, 307–344.

[39] Bai-Qing S., Yue Q., & Shan X. (2013). Improvement of Relational Two-Stage DEA Model under Fuzzy Chance Constraints. The 20th International Conference on Management Science and Engineerin*g*, 306–313. IEEE.

[40] Yousefi S., Soltani R., Farzipoor Saen R., & Pishvaee M. S. (2017). A Robust Fuzzy Possibilistic Programming for a New Network GP-DEA Model to Evaluate Sustainable Supply Chains. *Journal of Cleaner Production*, 166, 537–549.

[41] Zhou X., Luo R., Lev B., & Tu Y. (2017). Two-Stage Fuzzy DEA Models with Undesirable Outputs for Banking System. *International Conference on Management Science and Engineering Management*, 1604–1615. Springer, Cham.

[42] Peykani P., & Mohammadi E. (2018). Fuzzy Network Data Envelopment Analysis: A Possibility Approach. *The 3th International Conference on Intelligent Decision Science*, Iran.

[43] Zhou X., Xu Z., Yao L., Tu Y., Lev B., & Pedrycz W. (2018). A Novel Data Envelopment Analysis Model for Evaluating Industrial Production and Environmental Management System. *Journal of Cleaner Production*, 170, 773–788.

[44] Nasseri S. H., & Khatir M. A. (2019). Fuzzy Stochastic Undesirable Two-Stage Data Envelopment Analysis Models with Application to Banking Industry. *Journal of Intelligent & Fuzzy Systems*, 37(5), 7047–7057.

[45] Nosrat A., Sanei M., Payan A., Hosseinzadeh Lotfi F., & Razavyan S. (2019). Using Credibility Theory to Evaluate the Fuzzy Two-Stage DEA; Sensitivity and Stability Analysis. *Journal of Intelligent & Fuzzy Systems*, 37(4), 5777–5796.

[46] Roghaee N., Mohammadi E., & Varzgani N. (2020). Performance Evaluation and Ranking of Electricity Companies Using Fuzzy Network Data Envelopment Analysis: A Case Study of Iranian Regional Electricity Organisations. *International Journal of Management and Decision Making*, 19(4), 450–472.

[47] Chen R., & Xu Z. (2021). Efficiency evaluation of green supply chains based on fuzzy chance constrained three-stage DEA model. *IOP Conference Series*: *Earth and Environmental Science*, 770(1), 012039. IOP Publishing.

[48] Peykani P., Mohammadi E., & Emrouznejad A. (2021). An adjustable fuzzy chance-constrained network DEA approach with application to ranking investment firms. *Expert Systems with Applications*, 166, 113938.

[49] Pourbabagol H., Amiri M., Taghavifard M. T., & Hanafizadeh P. (2023). A new fuzzy DEA network based on possibility and necessity measures for agile supply chain performance evaluation: A case study. *Expert Systems with Applications*, 220, 119552.

[50] Premachandra I. M., Zhu J., Watson J., & Galagedera D. U. A. (2012). Best-performing US mutual fund families from 1993 to 2008: Evidence from a novel two-stage DEA model for efficiency decomposition. *Journal of Banking & Finance*, 36(12), 3302–3317.

[51] Galagedera D. U. A., Watson J., Premachandra I. M., & Chen Y. (2016). Modeling leakage in two-stage DEA models: An application to US mutual fund families. *Omega*, 61, 62–77.

[52] Premachandra I. M., Zhu J., Watson J., & Galagedera D. U. A. (2016). Mutual fund industry performance: a network data envelopment analysis approach. *Data Envelopment Analysis*, 165–228. Springer, Boston, MA.

[53] Sánchez-González C., Sarto J. L., & Vicente L. (2017). The efficiency of mutual fund companies: evidence from an innovative network SBM approach. *Omega*, 71, 114–128.

[54] Galagedera D. U. A., Roshdi I., Fukuyama H., & Zhu J. (2018). A new network DEA model for mutual fund performance appraisal: An application to U.S. equity mutual funds. *Omega*, 77, 168–179.

[55] Galagedera D. U. A. (2019). Modelling social responsibility in mutual fund performance appraisal: A two-stage data envelopment analysis model with non-discretionary first stage output. *European Journal of Operational Research*, 273(1), 376–389.

[56] Hsieh H. P., Tebourbi I., Lu W. M., & Liu N. Y. (2020). Mutual fund performance: the decision quality and capital magnet efficiencies. *Managerial and Decision Economics*, 41(5), 861–872.

[57] Galagedera D. U. A., Fukuyama H., Watson J., & Tan E. K. (2020). Do mutual fund managers earn their fees? new measures for performance appraisal. *European Journal of Operational Research*, 287(2), 653–667.

[58] Tsolas I. E. (2020). Precious metal mutual fund performance evaluation: a series two-stage DEA modeling approach. *Journal of Risk and Financial Management*, 13(5), 87.

[59] Fukuyama H., & Galagedera D. U. A. (2021). Value Extracting in Relative Performance Appraisal with Network DEA: An Application to US Equity Mutual Funds. *Data-Enabled Analytics: DEA for Big Data*, 263–297. Cham: Springer International Publishing.

[60] Peykani P., Emrouznejad A., Mohammadi E., & Gheidar-Kheljani J. (2022). A novel robust network data envelopment analysis approach for performance assessment of mutual funds under uncertainty. *Annals of Operations Research*, 1–27.

[61] Chen Y., Cook W. D., Li N., & Zhu J. (2009). Additive efficiency decomposition in two-stage DEA. *European Journal of Operational Research*, 196(3), 1170–1176.

[62] Koronakos G. (2019). A taxonomy and review of the network data envelopment analysis literature. *Machine Learning Paradigms: Applications of Learning and Analytics in Intelligent Systems*, 255–311.

[63] Charnes A., & Cooper W. W. (1962). Programming with Linear Fractional Functionals. *Naval Research Logistics Quarterly*, 9(3‐4), 181–186.

[64] Kao C., & Hwang S. N. (2008). Efficiency decomposition in two-stage data envelopment analysis: An application to non-life insurance companies in Taiwan. *European Journal of Operational Research*, 185(1), 418–429.

[65] Peykani P., Mohammadi E., Emrouznejad A., Pishvaee M. S., & Rostamy-Malkhalifeh M. (2019). Fuzzy data envelopment analysis: an adjustable approach. *Expert Systems with Applications*, 136, 439–452.

[66] Peykani P., Mohammadi E., Jabbarzadeh A., Rostamy-Malkhalifeh M., & Pishvaee M. S. (2020). A novel two-phase robust portfolio selection and optimization approach under uncertainty: A case study of Tehran stock exchange. *Plos One*, 15(10), e0239810. doi: 10.1371/journal.pone.0239810 33045010 PMC7549800

[67] Liu B. (2006). A survey of credibility theory. *Fuzzy Optimization and Decision Making*, 5, 387–408.

[68] Charnes A., & Cooper W. W. (1959). Chance-Constrained Programming. *Management Science*, 6(1), 73–79.

[69] Kao C. (2014). Network data envelopment analysis: A review. *European Journal of Operational Research*, 239(1), 1–16.

[70] Kao C. (2016). *Network Data Envelopment Analysis*: *Foundations and Extensions*. Springer.

[71] Ratner S. V., Shaposhnikov A. M., & Lychev A. V. (2023). Network DEA and its applications (2017–2022): A systematic literature review. *Mathematics*, 11(9), 2141.

[72] Izadikhah M., & Farzipoor Saen R. (2016). Evaluating sustainability of supply chains by two-stage range directional measure in the presence of negative data. *Transportation Research Part D: Transport and Environment*, 49, 110–126.

[73] Tavana M., Izadikhah M., Di Caprio D., & Farzipoor Saen R. (2018). A new dynamic range directional measure for two-stage data envelopment analysis models with negative data. *Computers & Industrial Engineering*, 115, 427–448.

[74] Omrani H., Emrouznejad A., Shamsi M., & Fahimi P. (2022). Evaluation of insurance companies considering uncertainty: A multi-objective network data envelopment analysis model with negative data and undesirable outputs. *Socio-Economic Planning Sciences*, 82, 101306.

[75] Fathollah Bayati M., & Sadjadi S. J. (2017). Robust network data envelopment analysis approach to evaluate the efficiency of regional electricity power networks under uncertainty. *Plos One*, 12(9), e0184103. doi: 10.1371/journal.pone.0184103 28953900 PMC5617154

[76] Zhou Z., Lin L., Xiao H., Ma C., & Wu S. (2017). Stochastic network DEA models for two-stage systems under the centralized control organization mechanism. *Computers & Industrial Engineering*, 110, 404–412.

[77] Ghaffari-Hadigheh A., & Lio W. (2020). Network data envelopment analysis in uncertain environment. *Computers & Industrial Engineering*, 148, 106657.

