# Supplementary material for: Malmquist productivity index for two-stage network systems under data uncertainty: A real-world case study
Source: PLoS One. 2024 Jul 18;19(7):e0307277. doi: 10.1371/journal.pone.0307277 (PMC11257370; doi:10.1371/journal.pone.0307277)
Supplement: S1 Appendix — (DOCX) [file pone.0307277.s001.docx]

**Appendix**

If we assume that the efficiency of the first stage carries more significance for the decision maker, efficiency change and technological change for first stage of the are obtained from Models (A1) to (A4):

| $\Theta_{k (\xi)}^{\text{S1} (t)}\left(I_{k}^{ t}, L_{k}^{ t}, C_{k}^{ t}, A_{k}^{ t}, O_{k}^{ t}\right)= \text{Max} ϒ$ | **(A1)** |
| --- | --- |
| $\begin{matrix} \text{S.t.} \sum_{y=1}^{Y} \left((2\xi) L_{yk}^{(2) t}+(1-2\xi) L_{yk}^{(3) t}\right)\lambda_{y} \\ +\sum_{g=1}^{G} \left((2\xi) C_{gk}^{(2) t}+(1-2\xi) C_{gk}^{(3) t}\right)\omega_{g}\geqϒ-\Gamma\Omega\end{matrix}$ | |
| $\begin{matrix} \sum_{y=1}^{Y} \left((2\xi-1) L_{yk}^{(1) t}+(2-2\xi) L_{yk}^{(2) t}\right)\lambda_{y} \\ +\sum_{g=1}^{G} \left((2\xi-1) C_{gk}^{(1) t}+(2-2\xi) C_{gk}^{(2) t}\right)\omega_{g}\geqϒ-\Gamma(1-\Omega) \end{matrix}$ | |
| $\sum_{p=1}^{P} \left((1-2\xi) I_{pk}^{(1) t}+(2\xi) I_{pk}^{(2) t}\right)\alpha_{p}\leq1+\Gamma\Omega$ |  |
| $\sum_{p=1}^{P} \left((2-2\xi) I_{pk}^{(2) t}+(2\xi-1) I_{pk}^{(3) t}\right)\alpha_{p}\leq1+\Gamma(1-\Omega)$ |  |
| $\begin{matrix} \sum_{y=1}^{Y} \left((1-2\xi) L_{ys}^{(1) t}+(2\xi) L_{ys}^{(2) t}\right)\lambda_{y}+\sum_{g=1}^{G} \left((1-2\xi) C_{gk}^{(1) t}+(2\xi) C_{gk}^{(2) t}\right)\omega_{g} \\ -\sum_{p=1}^{P} \left((2\xi) I_{ps}^{(2) t}+(1-2\xi) I_{ps}^{(3) t}\right)\alpha_{p}\leq\Gamma\Omega, \forall s \end{matrix}$ |  |
| $\begin{matrix} \sum_{y=1}^{Y} \left((2-2\xi) L_{ys}^{(2) t}+(2\xi-1) L_{ys}^{(3) t}\right)\lambda_{y}+\sum_{g=1}^{G} \left((2-2\xi) C_{gk}^{(2) t}+(2\xi-1) C_{gk}^{(3) t}\right)\omega_{g} \\ -\sum_{p=1}^{P} \left((2\xi-1) I_{ps}^{(1) t}+(2-2\xi) I_{ps}^{(2) t}\right)\alpha_{p}\leq\Gamma(1-\Omega) , \forall s \end{matrix}$ |  |
| $\begin{matrix} \sum_{q=1}^{Q} \left((1-2\xi) O_{qs}^{(1) t}+(2\xi) O_{qs}^{(2) t}\right)\beta_{q}-\sum_{g=1}^{G} \left((2\xi) C_{gs}^{(2) t}+(1-2\xi) C_{gs}^{(3) t}\right)\omega_{g} \\ -\sum_{x=1}^{X} \left((2\xi) A_{xs}^{(2) t}+(1-2\xi) A_{xs}^{(3) t}\right)\mu_{x}\leq\Gamma\Omega, \forall s \end{matrix}$ |  |
| $\begin{matrix} \sum_{q=1}^{Q} \left((2-2\xi) O_{qs}^{(2) t}+(2\xi-1) O_{qs}^{(3) t}\right)\beta_{q}-\sum_{g=1}^{G} \left((2\xi-1) C_{gs}^{(1) t}+(2-2\xi) C_{gs}^{(2) t}\right)\omega_{g} \\ -\sum_{x=1}^{X} \left((2\xi-1) A_{xs}^{(1) t}+(2-2\xi) A_{xs}^{(2) t}\right)\mu_{x}\leq\Gamma(1-\Omega) , \forall s \end{matrix}$ |  |
|  | |
|  | |
|  |  |
|  |  |
|  |  |
|  |  |

| $\Theta_{k (\xi)}^{\text{S1} (t+1)}\left(I_{k}^{ t+1}, L_{k}^{ t+1}, C_{k}^{ t+1}, A_{k}^{ t+1}, O_{k}^{ t+1}\right)= \text{Max} ϒ$ | **(A2)** |
| --- | --- |
| $\begin{matrix} \text{S.t.} \sum_{y=1}^{Y} \left((2\xi) L_{yk}^{(2) t+1}+(1-2\xi) L_{yk}^{(3) t+1}\right)\lambda_{y} \\ +\sum_{g=1}^{G} \left((2\xi) C_{gk}^{(2) t+1}+(1-2\xi) C_{gk}^{(3) t+1}\right)\omega_{g}\geqϒ-\Gamma\Omega\end{matrix}$ | |
| $\begin{matrix} \sum_{y=1}^{Y} \left((2\xi-1) L_{yk}^{(1) t+1}+(2-2\xi) L_{yk}^{(2) t+1}\right)\lambda_{y} \\ +\sum_{g=1}^{G} \left((2\xi-1) C_{gk}^{(1) t+1}+(2-2\xi) C_{gk}^{(2) t+1}\right)\omega_{g}\geqϒ-\Gamma(1-\Omega) \end{matrix}$ | |
| $\sum_{p=1}^{P} \left((1-2\xi) I_{pk}^{(1) t+1}+(2\xi) I_{pk}^{(2) t+1}\right)\alpha_{p}\leq1+\Gamma\Omega$ |  |
| $\sum_{p=1}^{P} \left((2-2\xi) I_{pk}^{(2) t+1}+(2\xi-1) I_{pk}^{(3) t+1}\right)\alpha_{p}\leq1+\Gamma(1-\Omega)$ |  |
| $\begin{matrix} \sum_{y=1}^{Y} \left((1-2\xi) L_{ys}^{(1) t+1}+(2\xi) L_{ys}^{(2) t+1}\right)\lambda_{y}+\sum_{g=1}^{G} \left((1-2\xi) C_{gk}^{(1) t+1}+(2\xi) C_{gk}^{(2) t+1}\right)\omega_{g} \\ -\sum_{p=1}^{P} \left((2\xi) I_{ps}^{(2) t+1}+(1-2\xi) I_{ps}^{(3) t+1}\right)\alpha_{p}\leq\Gamma\Omega, \forall s \end{matrix}$ |  |
| $\begin{matrix} \sum_{y=1}^{Y} \left((2-2\xi) L_{ys}^{(2) t+1}+(2\xi-1) L_{ys}^{(3) t+1}\right)\lambda_{y}+\sum_{g=1}^{G} \left((2-2\xi) C_{gk}^{(2) t+1}+(2\xi-1) C_{gk}^{(3) t+1}\right)\omega_{g} \\ -\sum_{p=1}^{P} \left((2\xi-1) I_{ps}^{(1) t+1}+(2-2\xi) I_{ps}^{(2) t+1}\right)\alpha_{p}\leq\Gamma(1-\Omega) , \forall s \end{matrix}$ |  |
| $\begin{matrix} \sum_{q=1}^{Q} \left((1-2\xi) O_{qs}^{(1) t+1}+(2\xi) O_{qs}^{(2) t+1}\right)\beta_{q}-\sum_{g=1}^{G} \left((2\xi) C_{gs}^{(2) t+1}+(1-2\xi) C_{gs}^{(3) t+1}\right)\omega_{g} \\ -\sum_{x=1}^{X} \left((2\xi) A_{xs}^{(2) t+1}+(1-2\xi) A_{xs}^{(3) t+1}\right)\mu_{x}\leq\Gamma\Omega, \forall s \end{matrix}$ |  |
| $\begin{matrix} \sum_{q=1}^{Q} \left((2-2\xi) O_{qs}^{(2) t+1}+(2\xi-1) O_{qs}^{(3) t+1}\right)\beta_{q}-\sum_{g=1}^{G} \left((2\xi-1) C_{gs}^{(1) t+1}+(2-2\xi) C_{gs}^{(2) t+1}\right)\omega_{g} \\ -\sum_{x=1}^{X} \left((2\xi-1) A_{xs}^{(1) t+1}+(2-2\xi) A_{xs}^{(2) t+1}\right)\mu_{x}\leq\Gamma(1-\Omega) , \forall s \end{matrix}$ |  |
|  | |
|  | |
|  |  |
|  |  |
|  |  |
|  |  |

| $\Theta_{k (\xi)}^{\text{S1} (t)}\left(I_{k}^{ t+1}, L_{k}^{ t+1}, C_{k}^{ t+1}, A_{k}^{ t+1}, O_{k}^{ t+1}\right)= \text{Max} ϒ$ | **(A3)** |
| --- | --- |
| $\begin{matrix} \text{S.t.} \sum_{y=1}^{Y} \left((2\xi) L_{yk}^{(2) t+1}+(1-2\xi) L_{yk}^{(3) t+1}\right)\lambda_{y} \\ +\sum_{g=1}^{G} \left((2\xi) C_{gk}^{(2) t+1}+(1-2\xi) C_{gk}^{(3) t+1}\right)\omega_{g}\geqϒ-\Gamma\Omega\end{matrix}$ | |
| $\begin{matrix} \sum_{y=1}^{Y} \left((2\xi-1) L_{yk}^{(1) t+1}+(2-2\xi) L_{yk}^{(2) t+1}\right)\lambda_{y} \\ +\sum_{g=1}^{G} \left((2\xi-1) C_{gk}^{(1) t+1}+(2-2\xi) C_{gk}^{(2) t+1}\right)\omega_{g}\geqϒ-\Gamma(1-\Omega) \end{matrix}$ | |
| $\sum_{p=1}^{P} \left((1-2\xi) I_{pk}^{(1) t+1}+(2\xi) I_{pk}^{(2) t+1}\right)\alpha_{p}\leq1+\Gamma\Omega$ |  |
| $\sum_{p=1}^{P} \left((2-2\xi) I_{pk}^{(2) t+1}+(2\xi-1) I_{pk}^{(3) t+1}\right)\alpha_{p}\leq1+\Gamma(1-\Omega)$ |  |
| $\begin{matrix} \sum_{y=1}^{Y} \left((1-2\xi) L_{ys}^{(1) t}+(2\xi) L_{ys}^{(2) t}\right)\lambda_{y}+\sum_{g=1}^{G} \left((1-2\xi) C_{gk}^{(1) t}+(2\xi) C_{gk}^{(2) t}\right)\omega_{g} \\ -\sum_{p=1}^{P} \left((2\xi) I_{ps}^{(2) t}+(1-2\xi) I_{ps}^{(3) t}\right)\alpha_{p}\leq\Gamma\Omega, \forall s \end{matrix}$ |  |
| $\begin{matrix} \sum_{y=1}^{Y} \left((2-2\xi) L_{ys}^{(2) t}+(2\xi-1) L_{ys}^{(3) t}\right)\lambda_{y}+\sum_{g=1}^{G} \left((2-2\xi) C_{gk}^{(2) t}+(2\xi-1) C_{gk}^{(3) t}\right)\omega_{g} \\ -\sum_{p=1}^{P} \left((2\xi-1) I_{ps}^{(1) t}+(2-2\xi) I_{ps}^{(2) t}\right)\alpha_{p}\leq\Gamma(1-\Omega) , \forall s \end{matrix}$ |  |
| $\begin{matrix} \sum_{q=1}^{Q} \left((1-2\xi) O_{qs}^{(1) t}+(2\xi) O_{qs}^{(2) t}\right)\beta_{q}-\sum_{g=1}^{G} \left((2\xi) C_{gs}^{(2) t}+(1-2\xi) C_{gs}^{(3) t}\right)\omega_{g} \\ -\sum_{x=1}^{X} \left((2\xi) A_{xs}^{(2) t}+(1-2\xi) A_{xs}^{(3) t}\right)\mu_{x}\leq\Gamma\Omega, \forall s \end{matrix}$ |  |
| $\begin{matrix} \sum_{q=1}^{Q} \left((2-2\xi) O_{qs}^{(2) t}+(2\xi-1) O_{qs}^{(3) t}\right)\beta_{q}-\sum_{g=1}^{G} \left((2\xi-1) C_{gs}^{(1) t}+(2-2\xi) C_{gs}^{(2) t}\right)\omega_{g} \\ -\sum_{x=1}^{X} \left((2\xi-1) A_{xs}^{(1) t}+(2-2\xi) A_{xs}^{(2) t}\right)\mu_{x}\leq\Gamma(1-\Omega) , \forall s \end{matrix}$ |  |
|  | |
|  | |
|  |  |
|  |  |
|  |  |
|  |  |

| $\Theta_{k (\xi)}^{\text{S1} (t+1)}\left(I_{k}^{ t}, L_{k}^{ t}, C_{k}^{ t}, A_{k}^{ t}, O_{k}^{ t}\right)= \text{Max} ϒ$ | **(A4)** |
| --- | --- |
| $\begin{matrix} \text{S.t.} \sum_{y=1}^{Y} \left((2\xi) L_{yk}^{(2) t}+(1-2\xi) L_{yk}^{(3) t}\right)\lambda_{y} \\ +\sum_{g=1}^{G} \left((2\xi) C_{gk}^{(2) t}+(1-2\xi) C_{gk}^{(3) t}\right)\omega_{g}\geqϒ-\Gamma\Omega\end{matrix}$ | |
| $\begin{matrix} \sum_{y=1}^{Y} \left((2\xi-1) L_{yk}^{(1) t}+(2-2\xi) L_{yk}^{(2) t}\right)\lambda_{y} \\ +\sum_{g=1}^{G} \left((2\xi-1) C_{gk}^{(1) t}+(2-2\xi) C_{gk}^{(2) t}\right)\omega_{g}\geqϒ-\Gamma(1-\Omega) \end{matrix}$ | |
| $\sum_{p=1}^{P} \left((1-2\xi) I_{pk}^{(1) t}+(2\xi) I_{pk}^{(2) t}\right)\alpha_{p}\leq1+\Gamma\Omega$ |  |
| $\sum_{p=1}^{P} \left((2-2\xi) I_{pk}^{(2) t}+(2\xi-1) I_{pk}^{(3) t}\right)\alpha_{p}\leq1+\Gamma(1-\Omega)$ |  |
| $\begin{matrix} \sum_{y=1}^{Y} \left((1-2\xi) L_{ys}^{(1) t+1}+(2\xi) L_{ys}^{(2) t+1}\right)\lambda_{y}+\sum_{g=1}^{G} \left((1-2\xi) C_{gk}^{(1) t+1}+(2\xi) C_{gk}^{(2) t+1}\right)\omega_{g} \\ -\sum_{p=1}^{P} \left((2\xi) I_{ps}^{(2) t+1}+(1-2\xi) I_{ps}^{(3) t+1}\right)\alpha_{p}\leq\Gamma\Omega, \forall s \end{matrix}$ |  |
| $\begin{matrix} \sum_{y=1}^{Y} \left((2-2\xi) L_{ys}^{(2) t+1}+(2\xi-1) L_{ys}^{(3) t+1}\right)\lambda_{y}+\sum_{g=1}^{G} \left((2-2\xi) C_{gk}^{(2) t+1}+(2\xi-1) C_{gk}^{(3) t+1}\right)\omega_{g} \\ -\sum_{p=1}^{P} \left((2\xi-1) I_{ps}^{(1) t+1}+(2-2\xi) I_{ps}^{(2) t+1}\right)\alpha_{p}\leq\Gamma(1-\Omega) , \forall s \end{matrix}$ |  |
| $\begin{matrix} \sum_{q=1}^{Q} \left((1-2\xi) O_{qs}^{(1) t+1}+(2\xi) O_{qs}^{(2) t+1}\right)\beta_{q}-\sum_{g=1}^{G} \left((2\xi) C_{gs}^{(2) t+1}+(1-2\xi) C_{gs}^{(3) t+1}\right)\omega_{g} \\ -\sum_{x=1}^{X} \left((2\xi) A_{xs}^{(2) t+1}+(1-2\xi) A_{xs}^{(3) t+1}\right)\mu_{x}\leq\Gamma\Omega, \forall s \end{matrix}$ |  |
| $\begin{matrix} \sum_{q=1}^{Q} \left((2-2\xi) O_{qs}^{(2) t+1}+(2\xi-1) O_{qs}^{(3) t+1}\right)\beta_{q}-\sum_{g=1}^{G} \left((2\xi-1) C_{gs}^{(1) t+1}+(2-2\xi) C_{gs}^{(2) t+1}\right)\omega_{g} \\ -\sum_{x=1}^{X} \left((2\xi-1) A_{xs}^{(1) t+1}+(2-2\xi) A_{xs}^{(2) t+1}\right)\mu_{x}\leq\Gamma(1-\Omega) , \forall s \end{matrix}$ |  |
|  | |
|  | |
|  |  |
|  |  |
|  |  |
|  |  |

Finally, efficiency change and technological change for second stage of the are obtained from Equations (A5) to (A8):

| $\begin{matrix} \Theta_{k (\xi)}^{\text{S2*} (t)}\left(I_{k}^{ t}, L_{k}^{ t}, C_{k}^{ t}, A_{k}^{ t}, O_{k}^{ t}\right)=\frac{\Theta_{k (\xi)}^{\text{N*} (t)}\left(I_{k}^{ t}, L_{k}^{ t}, C_{k}^{ t}, A_{k}^{ t}, O_{k}^{ t}\right)}{\Psi_{k (\xi)}^{\text{S2*} (t)}\left(I_{k}^{ t}, L_{k}^{ t}, C_{k}^{ t}, A_{k}^{ t}, O_{k}^{ t}\right)} \\ - \frac{\Psi_{k (\xi)}^{\text{S1*} (t)}\left(I_{k}^{ t}, L_{k}^{ t}, C_{k}^{ t}, A_{k}^{ t}, O_{k}^{ t}\right)\Theta_{k (\xi)}^{\text{S1*} (t)}\left(I_{k}^{ t}, L_{k}^{ t}, C_{k}^{ t}, A_{k}^{ t}, O_{k}^{ t}\right)}{\Psi_{k (\xi)}^{\text{S2*} (t)}\left(I_{k}^{ t}, L_{k}^{ t}, C_{k}^{ t}, A_{k}^{ t}, O_{k}^{ t}\right)} \end{matrix}$ | **(A5)** |
| --- | --- |

| $\begin{matrix} \Theta_{k (\xi)}^{\text{S2*} (t+1)}\left(I_{k}^{ t+1}, L_{k}^{ t+1}, C_{k}^{ t+1}, A_{k}^{ t+1}, O_{k}^{ t+1}\right)=\frac{\Theta_{k (\xi)}^{\text{N*} (t+1)}\left(I_{k}^{ t+1}, L_{k}^{ t+1}, C_{k}^{ t+1}, A_{k}^{ t+1}, O_{k}^{ t+1}\right)}{\Psi_{k (\xi)}^{\text{S2*} (t+1)}\left(I_{k}^{ t+1}, L_{k}^{ t+1}, C_{k}^{ t+1}, A_{k}^{ t+1}, O_{k}^{ t+1}\right)} \\ - \frac{\Psi_{k (\xi)}^{\text{S1*} (t+1)}\left(I_{k}^{ t+1}, L_{k}^{ t+1}, C_{k}^{ t+1}, A_{k}^{ t+1}, O_{k}^{ t+1}\right)\Theta_{k (\xi)}^{\text{S1*} (t+1)}\left(I_{k}^{ t+1}, L_{k}^{ t+1}, C_{k}^{ t+1}, A_{k}^{ t+1}, O_{k}^{ t+1}\right)}{\Psi_{k (\xi)}^{\text{S2*} (t+1)}\left(I_{k}^{ t+1}, L_{k}^{ t+1}, C_{k}^{ t+1}, A_{k}^{ t+1}, O_{k}^{ t+1}\right)} \end{matrix}$ | **(A6)** |
| --- | --- |

| $\begin{matrix} \Theta_{k (\xi)}^{\text{S2*} (t)}\left(I_{k}^{ t+1}, L_{k}^{ t+1}, C_{k}^{ t+1}, A_{k}^{ t+1}, O_{k}^{ t+1}\right)=\frac{\Theta_{k (\xi)}^{\text{N*} (t)}\left(I_{k}^{ t+1}, L_{k}^{ t+1}, C_{k}^{ t+1}, A_{k}^{ t+1}, O_{k}^{ t+1}\right)}{\Psi_{k (\xi)}^{\text{S2*} (t)}\left(I_{k}^{ t+1}, L_{k}^{ t+1}, C_{k}^{ t+1}, A_{k}^{ t+1}, O_{k}^{ t+1}\right)} \\ - \frac{\Psi_{k (\xi)}^{\text{S1*} (t)}\left(I_{k}^{ t+1}, L_{k}^{ t+1}, C_{k}^{ t+1}, A_{k}^{ t+1}, O_{k}^{ t+1}\right)\Theta_{k (\xi)}^{\text{S1*} (t)}\left(I_{k}^{ t+1}, L_{k}^{ t+1}, C_{k}^{ t+1}, A_{k}^{ t+1}, O_{k}^{ t+1}\right)}{\Psi_{k (\xi)}^{\text{S2*} (t)}\left(I_{k}^{ t+1}, L_{k}^{ t+1}, C_{k}^{ t+1}, A_{k}^{ t+1}, O_{k}^{ t+1}\right)} \end{matrix}$ | **(A7)** |
| --- | --- |

| $\begin{matrix} \Theta_{k (\xi)}^{\text{S2*} (t+1)}\left(I_{k}^{ t}, L_{k}^{ t}, C_{k}^{ t}, A_{k}^{ t}, O_{k}^{ t}\right)=\frac{\Theta_{k (\xi)}^{\text{N*} (t+1)}\left(I_{k}^{ t}, L_{k}^{ t}, C_{k}^{ t}, A_{k}^{ t}, O_{k}^{ t}\right)}{\Psi_{k (\xi)}^{\text{S2*} (t+1)}\left(I_{k}^{ t}, L_{k}^{ t}, C_{k}^{ t}, A_{k}^{ t}, O_{k}^{ t}\right)} \\ - \frac{\Psi_{k (\xi)}^{\text{S1*} (t+1)}\left(I_{k}^{ t}, L_{k}^{ t}, C_{k}^{ t}, A_{k}^{ t}, O_{k}^{ t}\right)\Theta_{k (\xi)}^{\text{S1*} (t+1)}\left(I_{k}^{ t}, L_{k}^{ t}, C_{k}^{ t}, A_{k}^{ t}, O_{k}^{ t}\right)}{\Psi_{k (\xi)}^{\text{S2*} (t+1)}\left(I_{k}^{ t}, L_{k}^{ t}, C_{k}^{ t}, A_{k}^{ t}, O_{k}^{ t}\right)} \end{matrix}$ | **(A8)** |
| --- | --- |
